# The mutualistic relationship between M2c macrophages of TGFβ1 induction and gastric cancer cells: the correlation between protective mechanisms in the tumor microenvironment and polarization of subtypes of cells

**DOI:** 10.7150/jca.97784

**Published:** 2025-02-03

**Authors:** Kaiqiang Meng, Jian Song, Fan Qi, Jiamin Li, Zhichao Fang, Liang Song, Shaonan Shi

**Affiliations:** 1 College of Traditional Chinese Medicine, Shaanxi University of Chinese Medicine, 712046, Shaanxi, China.; 2 College of Integrated Traditional Chinese and Western Medicine, Shaanxi University of Chinese Medicine, 712046, Shaanxi, China.; 3 Basic Medical College,Shaanxi University of Chinese Medicine, 712046, Shaanxi, China.

## Abstract

**Background:** Gastric cancer (GC) is one of the most common malignant tumors worldwide, with fast metastasis and high mortality rate. GC cells and tumor immune microenvironment exhibit high heterogeneity. Multiple pieces of evidence suggest that TGFβ1 intervenes in the tumor microenvironment, immune cells and GC prognosis. The aim of this study is to comprehensively investigate the functional intervention of macrophage polarization subtypes on gastric cancer cell lines in the GC tumor microenvironment, providing valuable insights into tumor microenvironment research and potential targets for treatment strategies.

**Methods:** TCGA database and multiple GEO datasets were used to validate the role of TGFβ1 in cancer prognosis, immune infiltration and subtype macrophage polarization. Construct different subtypes of macrophages and establish cell co culture systems using Transwell chambers. Enzyme linked immunosorbent assay (ELISA), western blotting (WB) and reverse transcription quantitative polymerase chain reaction (RT-qPCR) were used to verify the changes in the metastatic function and defense mechanism of gastric cancer cells (Hgc27 and MKN45) in different co culture systems. Further analyze the effect of gastric cancer cell metabolites on macrophage subtype polarization.

**Results:** TGFβ1 was highly expressed in GC tissues, highly expressed TGFβ1 could reduce the survival time of GC patients. The GC immune infiltration results confirmed the correlation between TGFβ1 and M2 macrophages. The GEO dataset results of gastric cancer at different stages showed that some M2 macrophage markers showed consistent changes with TGFβ1. The WB, ELISA and RT-qPCR have identified TGFβ1-induced polarization of M2c macrophages, most biomarkers are associated with M2c. M2c macrophages can enhance cell migration and function, can inhibit ferroptosis in gastric cancer cells, endowing them with stronger special environmental resistance. Gastric cancer cells tend to polarize towards M2 macrophages, with M2c being the main M2 subtype of macrophages.

**Conclusion:** In conclusion, our study reveals a mutually beneficial symbiotic relationship between M2c macrophages and cancer cells in the microenvironment of gastric cancer tumors. TGFβ1 promotes the production of M2c macrophages, which enhance the function and ferroptosis resistance of gastric cancer cells. Gastric cancer cells provide the material basis for M2c macrophage polarization. This new evidence may provide new insights into developing more effective targeted therapies for gastric cancer to combat the formation of immune escape and metastasis in gastric cancer.

## Introduction

Gastric cancer (GC) is the fifth most commonly diagnosed cancer worldwide and the fourth most common cause of cancer death 1. GC urgently needs effective diagnostic biomarkers and potential therapeutic targets 2, 3 to reverse the dangerous situation faced by GC patients 4. Most GC patients have a poor prognosis 5, 6, mainly due to late diagnosis and poor treatment outcomes 7. The TGFβ family seems to intervene in the progression of gastric cancer through multiple pathways. TGFβ1 plays an important role in the TGFβ family and has a significant impact on cell proliferation, growth and immune functions. At the same time, TGFβ1 plays a dual role in cancer development 8 and can transition from tumor suppressor to tumor enhancer in the later stages of cancer 9.

Tumor microenvironment (TME) is an important component of tumor tissue. More and more evidence confirms that TME has significant clinical and pathological significance in predicting outcomes and treatment 10. The TME of GC is composed of other cellular components such as extracellular matrix, immune cells and cancer cells 11. Macrophages are an important component of the GC TME, macrophages that infiltrate the TME are called tumor associated macrophages 12, 13, namely M1 type with anticancer function and M2 type with tumor promoting function 14. The main function of macrophages is to engulf foreign antigens while maintaining iron balance in body tissues 15. Surprisingly, malignant cells in TME can evade the harmful effects of iron overload and require a large amount of these reactive ions to proliferate 16. TGFβ1 has been shown to affect M2 macrophages in various cancer TMEs 17, 18. This induction is different from the classical IL-4 induction method, as different induction pathways affect the proportion of subtypes (M2a, M2b, M2c, M2d) in M2 macrophage clusters 19. Subtype changes in M2 macrophage clusters directly affect the metabolic products of M2 clusters, and TGFβ1 is closely associated with M2c macrophage subtypes 20.

Iron induced cell death is a cell death mode that relies on the accumulation of iron dependent lipid peroxides 21. Glutathione peroxidase 4 (GPX4) is a key regulator of iron overload 22, which uses reduced glutathione to convert lipid hydroperoxides into lipid alcohols, thereby reducing lipid peroxidation and inhibiting iron overload 23, 24. The increased expression of GPX4 in tumors is significantly correlated with tumor occurrence and metastasis. The high concentration of antioxidants in cancer cells is a significant obstacle in cancer radiotherapy and chemotherapy 25. TGFβ1 intervention in the occurrence of ferroptosis in various diseases, including cancer 26, 27, has become a research hotspot in the study of ferroptosis in diseases 28, 29.

Resistance to cell death is an important characteristic of cancer, and the high mortality rate and poor prognosis of GC indicate stronger resistance in TME and resistance to GC treatment 30. TGFβ1 seems to be an important bridge between ferroptosis in GC cells and polarization of M2 macrophages, and is involved in the key link of ferroptosis in TME 31. To explore these issues, we used various public data platforms such as TCGA database and GEO database to systematically discuss the role of TGFβ1 in cancer prognosis, immune infiltration and polarization of subtypes of macrophages. TGFβ1-induced M2c subtype macrophages have important interactions with gastric cancer cells (Hgc27 and MKN45) in terms of metastasis function and ferroptosis resistance and gastric cancer cells (Hgc27 and MKN45) provide important material basis for M2c macrophage polarization. Studying the effect of macrophage polarization subtypes on the functional intervention of gastric cancer cell lines in the GC TME provides valuable insights into TME research and potential targets for treatment strategies.

## Methods

### Microarray data processing

The microarray dataset of Stomach adenocarcinoma patients was obtained from the Gene Expression Omnibus (GEO) database (http://www.ncbi.nlm.nih.gov/geo/). The Cancer Genome Atlas (TCGA) database (https://portal.gdc.cancer.gov/). Extracted from within. Six microarray gene expression datasets (GSE65801 32, GSE84787 33, GSE103236 34, GSE70394 35, GSE21328 36 and GSE146895) were obtained from the GEO database. Detailed description of GEO dataset in Supplementary File Table S1. Detailed description of TCGA dataset in Supplementary File Table S3.

### Survival curve

The "survival" R package (version 4.0.2) was used to plot the Kaplan-Meier (KM) survival curve to analyze the relationship between DEG expression and overall survival in GC patients. P <0.05 was considered statistically significant.

### ROC analysis

Using a visualization tool (https://hiplot.com.cn/basic/roc) to perform multivariate modeling on TGFβ1 to identify biomarkers with high sensitivity and specificity for prognostic diagnosis. Randomly classify the sample set, using one data set as the training set and the other as the validation sample. Plot the characteristic curve and calculate the area under the curve (AUC) to evaluate the performance of each model, using the R package 'pROC'.

### Immune cell infiltration analysis

For the correlation study between differentially expressed genes and immune infiltration levels, we utilized the TIMER database (https://cistrome.shinyapps.io/timer/) to evaluate the differences in the infiltration of 22 immune cell types between the low-risk group and the high-risk group. Moreover, we used CIBERSORT (https://cibersort.stanford.edu/about.php) in combination with the TIMER database to estimate the proportion of immune cells in tumor tissues. And we visualized the correlation results between the main variables and the immune infiltration matrix data using the "ggplot2" package.

### Cell lines

Human acute monocytic leukemia cell line THP-1, gastric cancer cell lines (Hgc27 and MKN45) and human gastric mucosal cells GES1 and MC (as the non cancer control group) were purchased from the National Collection of Authenticated Cell Cultures (ShaanXi, China). The cells were cultured in DMEM medium (Boster) with 10% fetal bovine serum (Boster), 100 U/mL penicillin and 100 μg/mL streptomycin (Gibco), all cells under the condition of 5% CO and 37 °C.

### Cell Counting Kit-8 (CCK-8) assay for cell proliferation

Cells were seeded into 96-well plates at a density of 5,000 cells per well. After the cells grew stably, different concentrations of small molecule compounds (LPS, IL-4, TGFB1, RSL3 and Fer-1) were added for incubation according to the protocol. At the planned detection time points, the CCK-8 working solution was prepared at a ratio of 1:10, and 100 μL of basic medium was added to each well. After incubation at 37 °C for 40 minutes, the optical density (OD) value at 450 nm was measured using a microplate reader (Bio-Rad 680, Bio-Rad). The average OD values of each group were used to reflect the cell proliferation.

### Reverse transcription quantitative polymerase chain reaction (RT-qPCR) assay

TRIzol® Plus RNA Purification Kit (Invitrogen, Carlsbad, CA, USA) was used to perform total RNA extraction. First-strand cDNA was synthesized using SuperScript™ III First-strand Synthesis SuperMix for RT-qPCR (Invitrogen, Carlsbad, CA, USA). cDNA was then synthesized using Power SYBR® Green PCR Master Mix Kit (Applied Biosystems, USA) for real-time fluorescent quantitative PCR. Seven different substrates (CD86, iNOS, CD206, IL1R2, CD163 and TGFB) were used, primer related data is provided in Supplementary Data Table S2.

### Immunosorbent assay (ELISA)

Gently wash macrophages under different conditions with pre cooled PBS, then digest them with trypsin, centrifuge at 1000 ×g for 5 minutes, and collect the cells. Wash the collected cells three times with pre cooled PBS. Add 100 μL of cell lysis buffer (containing phosphatase inhibitor and protease inhibitor) to every 1 million cells, thoroughly lyse on ice, centrifuge at 14000g for 5 minutes, and take the supernatant. Operate according to the manufacturer's instructions using the Human TNF ELISA Kit (Boster, EK0525), Human IL-10 ELISA Kit (Boster, EK0416) and Human TGFB1 ELISA Kit (Boster, EK0513). Read the absorbance at 450 nm using an enzyme-linked immunosorbent assay reader.

### Western blotting (WB) assay

The expression level of ferroptosis key protein of gastric cancer cell line Hgc27, MKN45 and AGS was confirmed by protein blotting. Cells were treated with RIPA lysis buffer (Fdbio, #FD009) containing phosphatase inhibitors and protease inhibitors. Protein concentration was detected using BCA Protein Assay Kit (Boster, AR0146). Equivalent proteins were then separated by 10% Tris-Tricine SDS-PAGE and transferred to polyvinylidene difluoride (PVDF) membranes. After 2 h of closure with skimmed milk in TBST, the membranes were analyzed using TNF-α (Boster, A00002-5), IL-10 (Boster, A00021-2), TGFβ1 (Boster, BM4876), Smad2 (Immunoway, YP1185), p-Smad2 (Bioss, bs-20341R), Smad3 (Immunoway, YT4334), p-Smad3 (Bioss, bs-5235R), E-cadherin (Immunoway, YM0207), N-cadherin (Immunoway, YM4937), Vimentin (Immunoway, YT4880), GAPDH (Boster, BA2913), GPX4 (Boster, BM5231), FSP1 (Proteintech, #20886-1-AP), DHODH (Boster, M04035-1) and SLC7A11 (Boster, BM5318) were incubated overnight at 4°C. The membranes were subsequently washed with Tris-buffered saline containing Tween and then incubated with the corresponding coupled anti-rabbit (anti-mouse) antibodies for 1 hour at 37 °C. Finally, the bands on the membrane were visualized using a ChemiDoc™ imaging system.

### Detection of oxidative stress and antioxidant enzymes

We measured the levels of malondialdehyde (MDA), a product of oxidative stress, as well as the levels of the antioxidant enzymes superoxide dismutase (SOD) and glutathione (GSH) in gastric cancer cells. Gastric cancer cells were treated with 5 times the volume of cell lysis buffer and left on ice for 20 minutes. Then, the centrifuge was set to rotate at 3,000 revolutions per minute for 10 minutes and the supernatants were collected. According to the manufacturer's instructions, the MDA, SOD and GSH were measured using detection kits (produced by Nanjing Jiancheng Bioengineering Institute, China).

### Migration and invasion

The migratory and invasive abilities of cells were detected using 6-well Transwell chambers with a pore size of 8.0 µm. For the cell invasion experiment, matrix gel was added to the upper chamber of the Transwell chamber in advance and left to stand for 12 hours before starting the experiment, while no such pre-operation was required for the cell migration experiment. After the migration or invasion experiment in which the cells were cultured in the medium containing 10% fetal bovine serum for a certain period of time, the cells in the lower chamber of the Transwell chamber were fixed with 4% paraformaldehyde for 10 minutes, washed three times with PBS, then stained with 0.5% crystal violet for 30 minutes. Pictures were taken under a microscope and quantitative analysis was carried out.

### Scratch wound healing assay

The number of cells cultured normally in 6-well plates reached ≥90% and grew to confluence. Use a sterile pipette tip to make a uniform scratch in the center of the cell layer. Photographs were taken under a microscope at fixed time points after scratching (0 h, 12 h and 24 h), and the scratch distance was estimated using Image J software.

### Assessment of Mitochondrial Membrane Potential (MMP)

The mitochondrial probe 5,5',6,6'-tetrachloro-1,1',3,3'-tetraethylbenzimidazolylcarbocyanine iodide (JC-1) is a lipophilic cationic dye that exhibits green fluorescence (JC-1 monomers). The JC-1 dye can accumulate in normal mitochondria and form red fluorescent complexes (JC-1 aggregates), and the fluorescence intensity can be used to monitor changes in mitochondrial membrane potential. The mitochondrial membrane potential detection kit (produced by Beyotime, China) was used to detect the fluorescence intensity of JC-1. The JC-1 working solution was added to the cell culture medium and incubated at 37 °C for 20 minutes. The fluorescence intensity was obtained using a fluorescence microscope.

### DEGs identification

GEO2R (https://www.ncbi.nlm.nih.gov/geo/ geo2r/) is an interactive web tool for identifying differentially expressed genes (DEGs) of two or more groups of samples (GEO series with gene symbol) 37. The differentially expressed genes between gastric cancer induction group and different types of macrophages (M0, M1 and M2) are identified by GEO2R and set as the cut-off criteria 37. The Venn diagram is used to obtain the co-expressed genes (up-regulated and down-regulated) of the two expression profiles, and the heat map of the top 50 differentially expressed genes is plotted 38.

### GO function and KEGG pathway enrichment analysis of differentially expressed genes

G Gene Ontology (GO) analysis is an effective method for identifying the characteristic biological properties of transcriptional data or high-throughput genomes (annotating genes and gene products) 39. The Kyoto Encyclopedia of Genes and Genomes (KEGG) is a database covering biological pathways, diseases, drugs and chemicals. Use the online tool of the functional annotation tool (DAVID, https://david.ncifcrf.gov/) to analyze differentially expressed genes at the functional level.

### Statistical analysis

Statistical analyses mainly rely on GraphPad Prism 8 and SPSS 26.0. ImageJ software is mainly used for the quantification of protein fluorescence intensity and some affected images. Kaplan-Meier survival curves are drawn, and multivariate survival analysis is conducted and the Cox proportional hazards model is used. Data are presented as mean ± SD. One-way ANOVA is used for comparisons among multiple groups, while the Student's t-test is used for two-group comparisons. A p-value of < 0.05 is considered statistically significant.

## Results

### Prognostic results of gastric adenocarcinoma suggest that TGFβ1 has great potential

**Figure 1:**
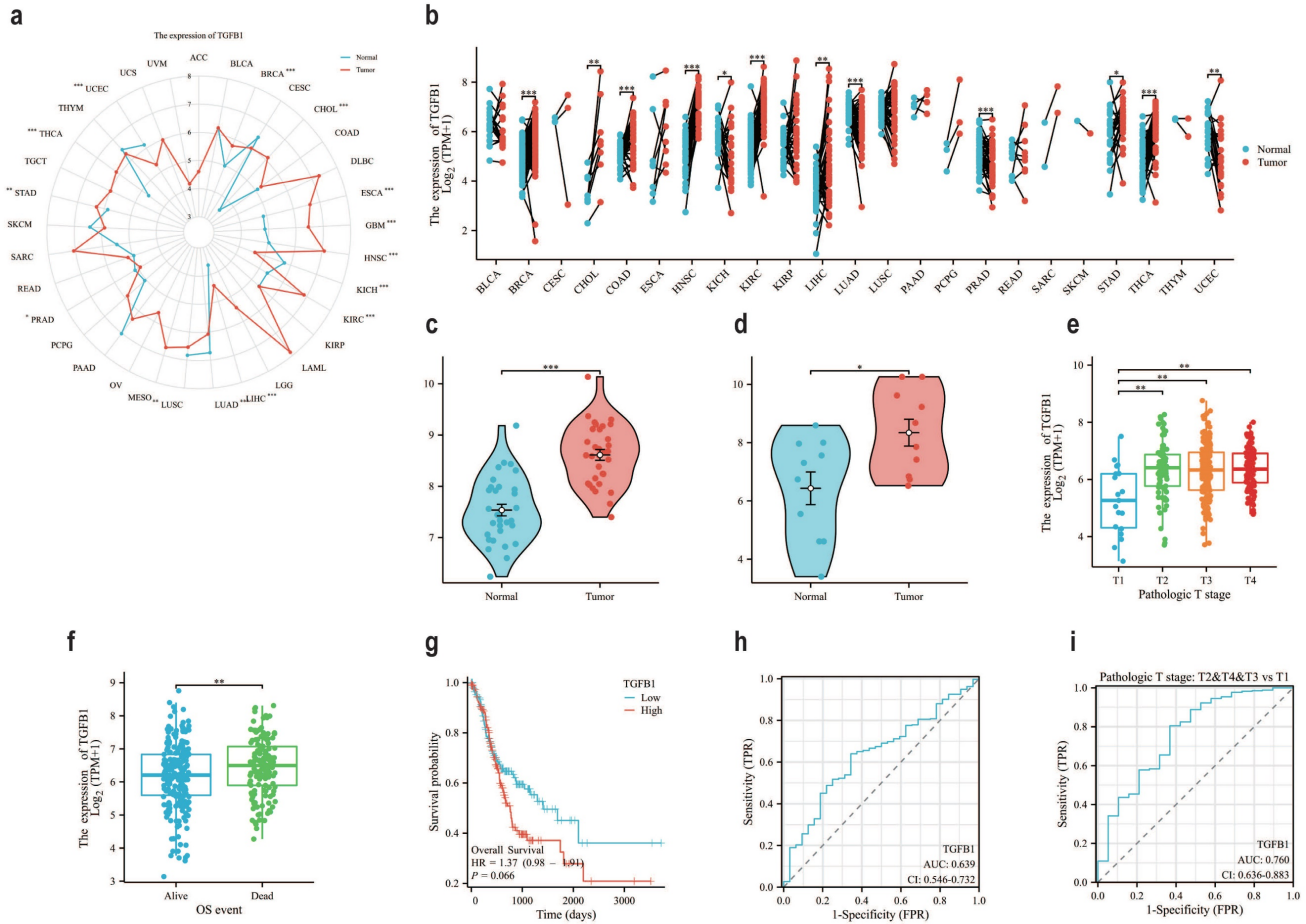
The prognosis of gastric adenocarcinoma suggests that TGFβ1 has great potential. **a** The pan cancer expression of TGFβ1 in the TCGA database. **b** The pan cancer expression of TGFβ1 in paired samples in the TCGA database. **c** The expression of TGFβ1 in the GSE65801 dataset. **d** The expression of TGFβ1 in the GSE84787 dataset. **e** T staging results of TGFβ1. **f** The differential expression of TGFβ1 in the quality of life of different patients. **g** Comparing the prognosis between high and low TGFβ1 groups based on the g KM survival curve. **h** Analyze the predictive accuracy and efficacy of TGFβ1 in GC queue diagnosis. **i** Diagnostic prediction of TGFβ1 in different T stages of GC queue. *p<0.05, **p<0.01, ***p<0.001.

In the TCGA database, compared with normal tissues of GC, the expression level of TGFβ1 in tumor samples was significantly increased (Fig. 1a), the same results were also observed in paired sample data of pan cancer data (Fig. 1b). It was also confirmed in two datasets of GEO database (GSE84787, GSE65801) that TGFβ1 showed higher expression in cancer tissues (Fig. 1c-d). Further analysis of the correlation between TGFβ1 expression and clinical pathological parameters in GC patients revealed significant differences in TGFβ1 expression levels between T1 and T2-T4 stages (Fig. 1e). Prognostic results showed high expression of TGFβ1 in deceased patients (Fig. 1f). According to the KM plot, the overall survival period of GC cases with low TGFβ1 expression was higher than that of GC cases with high expression (Fig. 1g). The diagnostic ROC was used to analyze the predictive accuracy of TGFβ1, the ROC curve showed AUC=0.639, indicating that TGFβ1 has certain diagnostic value in predicting outcomes (Fig. 1h). TGFβ1 showed a significant improvement in diagnostic prediction at different T stages (Fig. 1i).

### The association between TGFβ1 and the immune microenvironment associated with GC tumors

**Figure 2:**
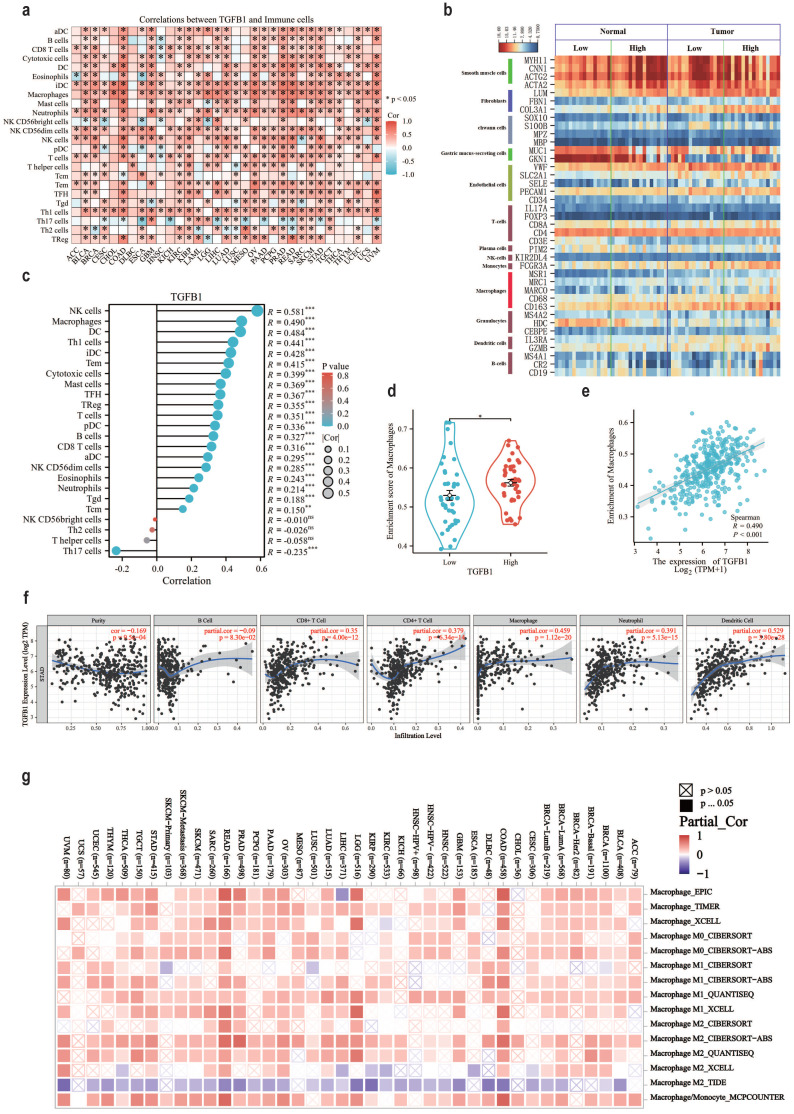
The association between TGFβ1 and the immune microenvironment associated with GC tumors. **a** TGFβ1 pan cancer status and tumor associated immune cell correlation heatmap in TCGA database. **b** The expression of cell markers in the GSE65801 dataset. **c** GC tumor associated immune cells and TGFβ1 correlation bar chart. **d** Box plot of the correlation between TGFβ1 differential expression group and macrophages. **e** Scatter plot showing the association between TGFβ1 and macrophages. **f** The correlation results between TGFβ1 and GC immune infiltrating cells in TIMER database. **g** Correlation heatmap between TGFβ1 and macrophage subtypes. *p<0.05, **p<0.01, ***p<0.001.

The pan cancer status and tumor associated immune cell correlation heatmap results of TGFβ1 in the TCGA database suggest that macrophages, DCs and NK cells are immune cells highly associated with TGFβ1 in GC (Fig. 2a). The stick chart results of GC tumor associated immune cells and TGFβ1 correlation showed that NK cells (R=0.581), macrophages (R=0.490) and DCs (R=0.484) were highly correlated immune cells in GC (Fig. 2c). Clean the GSE65801 data, assign type labels and sort them according to the expression level of TGFβ1. Smooth muscle cell markers are highly expressed in gastric adenocarcinoma and adjacent tissues, while gastric mucus screening cell markers are only highly expressed in adjacent tissues (Fig. 2b). The macrophage marker CD163 showed significant differences and was highly expressed in gastric adenocarcinoma tissue samples, with a trend consistent with TGFβ1, but other macrophage markers did not show the same trend (Fig. 2b). To further clarify the association between macrophages and TGFβ1 in GC, we compared the correlation between TGFβ1 differential expression group and macrophages, demonstrated the correlation between TGFβ1 differential expression group and macrophages (Fig. 2d). In GC, TGFβ1 was linearly correlated with macrophages (R=0.490) (Fig. 2e). The correlation between TGFβ1 expression and immune infiltration levels was validated using the TIMER database, the results showed a correlation between macrophages and TGFβ1 (Fig. 2f). Tumor associated macrophages can be classified into M1 and M2 based on functional differences. The association between TGFβ1 and macrophage subtypes showed that M2 macrophages were closely related to TGFβ1 in GC (Fig. 2g).

### There is a close correlation between TGFβ1 and polarization of M2c macrophages

**Figure 3:**
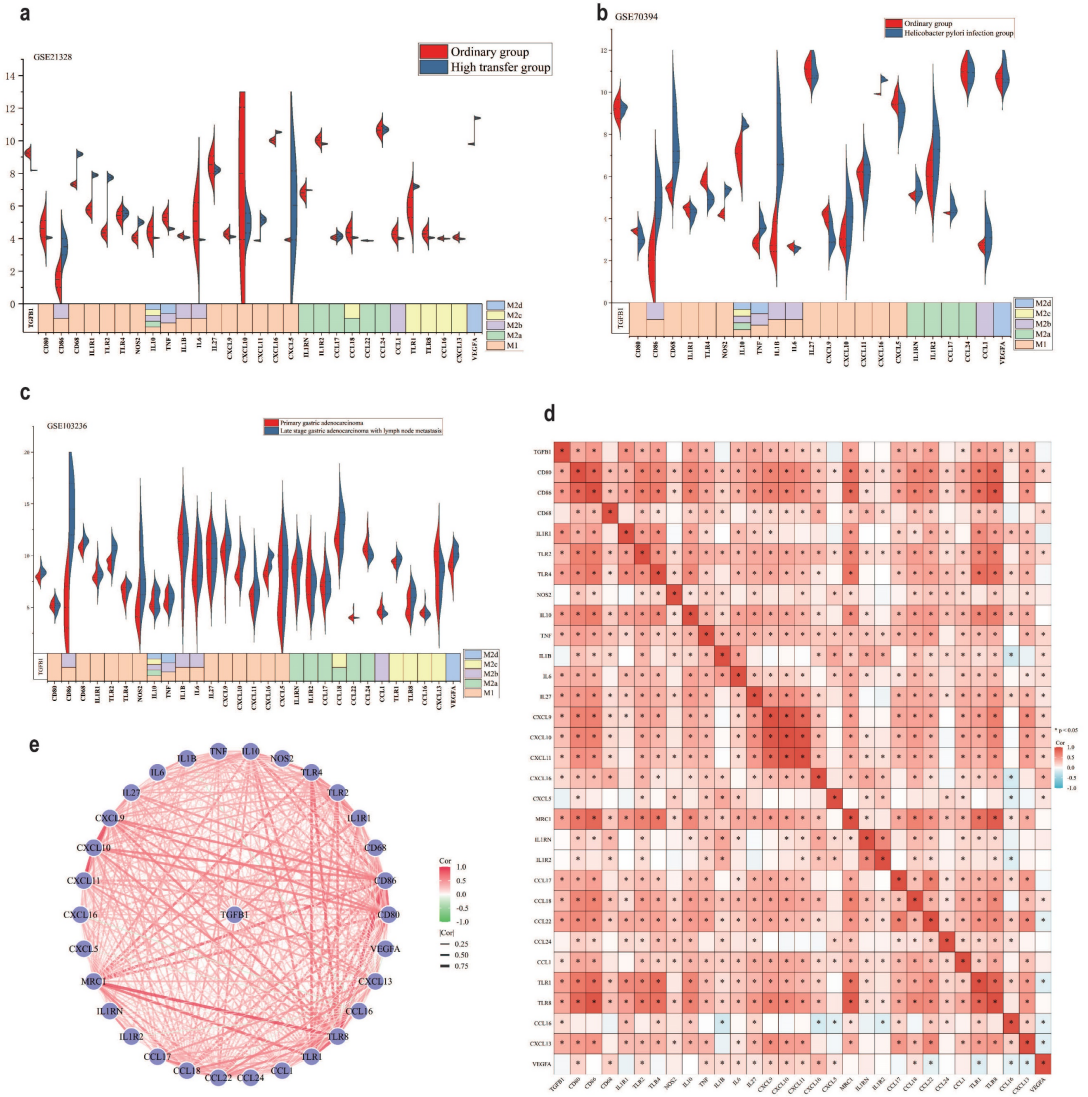
There is a close correlation between TGFβ1 and polarization of M2c macrophages. **a** The expression results of different biomarkers in the GSE21328 dataset. **b** The expression results of different biomarkers in the GSE70394 dataset. **c** The expression results of different biomarkers in the GSE103236 dataset. **d** Heat map of the association between TGFβ1 and different subtypes of macrophages. **e** The relationship between TGFβ1 and different subtypes of macrophage marker networks. *p<0.05, **p<0.01, ***p<0.001.

In the TME, different subtypes of M2 macrophages (M2a, M2b, M2c and M2d) (Different subtypes of macrophage markers are shown in Table S4) participate in regulating various immune escape processes, thereby promoting cancer development 40, 41. To further clarify the association between M2 macrophage subtypes and TGFβ1, we investigated the expression of different subtypes of macrophage markers in different GC datasets. The results of the GSE21328 dataset showed that compared with normal cell lines, the highly metastatic cell line had low expression of TGFβ1, the M2c macrophage markers TLR8, CCL16 and CXCL13 showed the same trend (Fig. 3a). Helicobacter pylori is a recognized pathogenic factor for gastric cancer, it promotes the further development of gastric cancer. The results of the GSE70394 dataset showed that compared with normal cell lines, Helicobacter pylori infected cell lines had low expression of TGFβ1, high expression of most M1 macrophage markers, high expression of IL-10, an inflammatory factor influenced by M2c (Fig. 3b). The results of the GSE103236 dataset showed that compared with primary GC, advanced GC with lymph node metastasis had high expression of TGFβ1 and high expression of M2c macrophage markers TLR1 and TLR8 (Fig. 3c). The heatmap results of the association between TGFβ1 and different subtypes of macrophages showed varying degrees of correlation between TGFβ1 and M2c macrophage markers (TLR1, TLR8, CCL16, and CXCL13) (Fig. 3d). Similarly, the STRING database analyzed the relationship between TGFβ1 and different subtypes of macrophage marker networks, the results showed varying degrees of correlation between TGFβ1 and M2c macrophage markers (TLR1, TLR8, CCL16, and CXCL13) (Fig. 3e).

### Different inducers promote polarization of different subtypes of macrophages

**Figure 4:**
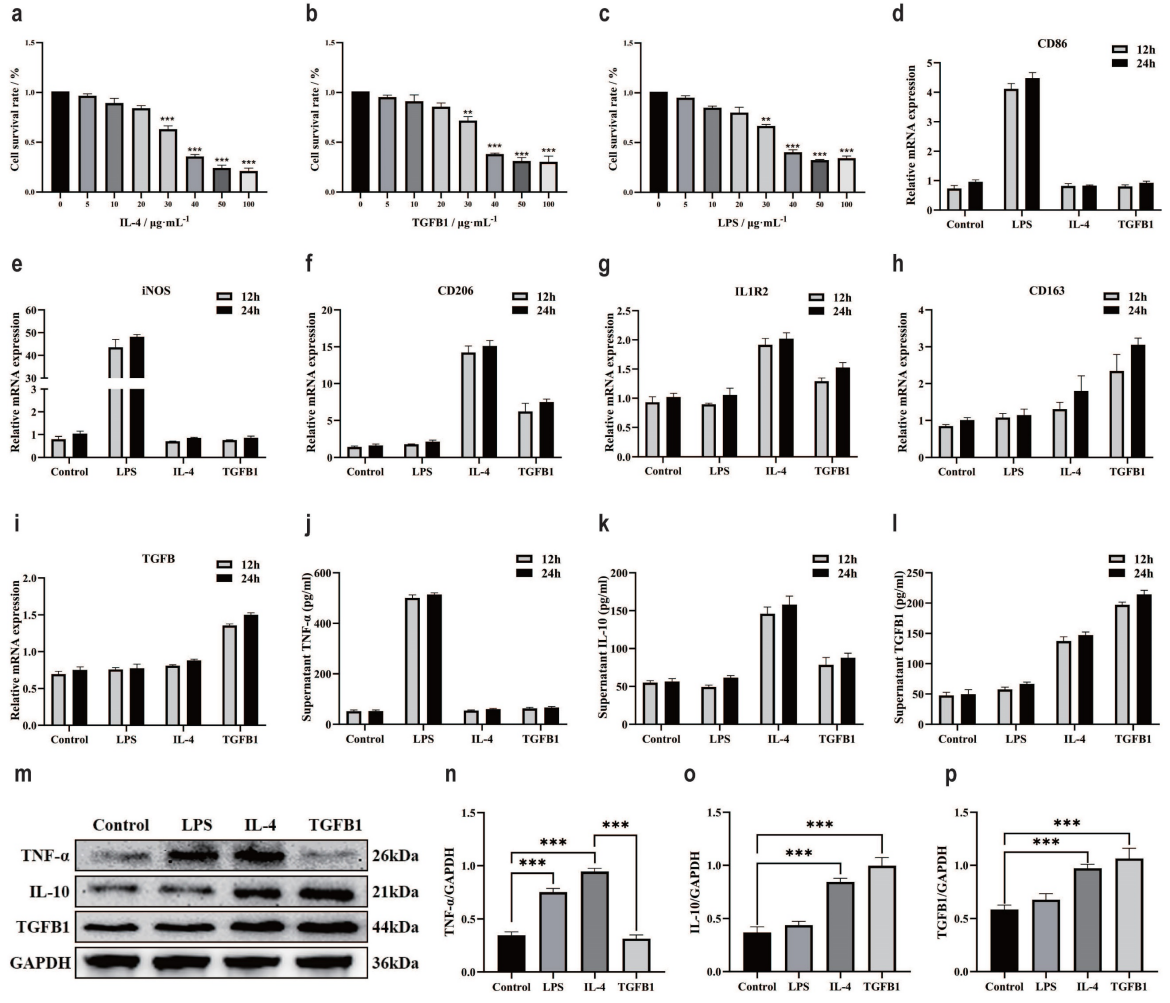
Different inducers promote polarization of different subtypes of macrophages. **a** The CCK-8 method was used to detect the survival of M0 macrophages after 24 h of IL-4 intervention. **b** The CCK-8 method was used to detect the survival of M0 macrophages after 24 h of intervention with TGFβ1. **c** The CCK-8 method was used to detect the survival of M0 macrophages after LPS intervention for 24 h. **d** RT-qPCR was used to detect the expression of CD86 mRNA. **e** RT-qPCR was used to detect the expression of iNOS mRNA. **f** RT-qPCR was used to detect the expression of CD206 mRNA. **g** RT-qPCR was used to detect the expression of IL1R2 mRNA. **h** RT-qPCR was used to detect the expression of CD163 mRNA. **i** RT-qPCR was used to detect the expression of TGFβ mRNA. **j** ELISA detects TNF - α levels. **k** ELISA was used to detect IL-10 levels. **l** ELISA detects the content of TGFβ1. **m** The WB results of different interventions on histone expression. **f** TNF-α protein expression results. **f** IL-10 protein expression results. **f** TGFβ1 protein expression results. *p<0.05, **p<0.01, ***p<0.001.

Macrophages are a large group of immune cells 42. In the TME, different subtypes of macrophages participate in different mechanisms of regulation 43. At present, it is clear that the polarization of macrophages is intervened by inducers, such as LPS inducing M1, IL-4 inducing M2a, and TGFβ1 seemingly inducing M2c 44, 45. Intervention of M0 macrophage polarization with small molecule compounds. The CCK-8 method was used to detect the effect of different small molecule compounds on the survival rate of M0 macrophage cells. After 24 hours of intervention, the results showed that the optimal intervention concentration for IL-4 was 20 μg/mL (Fig. 4a), TGFβ1 was 20 μg/mL (Fig. 4b), and LPS was 20 μg/mL (Fig. 4c). RT-qPCR was used to verify the polarization of macrophages by small molecule compounds. CD86 mRNA results showed a significant increase in LPS intervention group (Fig. 4d), iNOS mRNA results showed a significant increase in LPS intervention group (Fig. 4e), CD206 mRNA results showed a significant increase in IL-4 intervention group and a significant increase in TGFβ1 intervention group (Fig. 4f), IL1R2 mRNA results showed a significant increase in IL-4 intervention group and a significant increase in TGFβ1 intervention group (Fig. 4g), CD163 mRNA results showed a significant increase in TGFβ1 intervention group and a slight increase in IL-4 intervention group (Fig. 4h), TGFβ mRNA results showed a significant increase in TGFβ1 intervention group and a slight increase in IL-4 intervention group (Fig. 4i).). ELISA examined markers of macrophage polarization, TNF-α results showed a significant increase in the LPS intervention group (Fig. 4j). IL-10 results showed a significant increase in the IL-4 intervention group and a significant increase in the TGFβ1 intervention group (Fig. 4k). TGFβ1 results showed a significant increase in the TGFβ1 intervention group and a significant increase in the IL-4 intervention group (Fig. 4l). The protein expression of markers for macrophage polarization was validated by WB (Fig. 4m), the results of TNF-α protein expression showed a significant increase in the LPS intervention group and IL-4 intervention group (Fig. 4n). The protein expression of IL-10 showed a significant increase in the IL-4 intervention group and TGFβ1 intervention group (Fig. 4o), and the protein expression of TGFβ1 showed a significant increase in the IL-4 intervention group and TGFβ1 intervention group (Fig. 4p).

### M2 subtype macrophages have an impact on the function of gastric cancer cells

**Figure 5:**
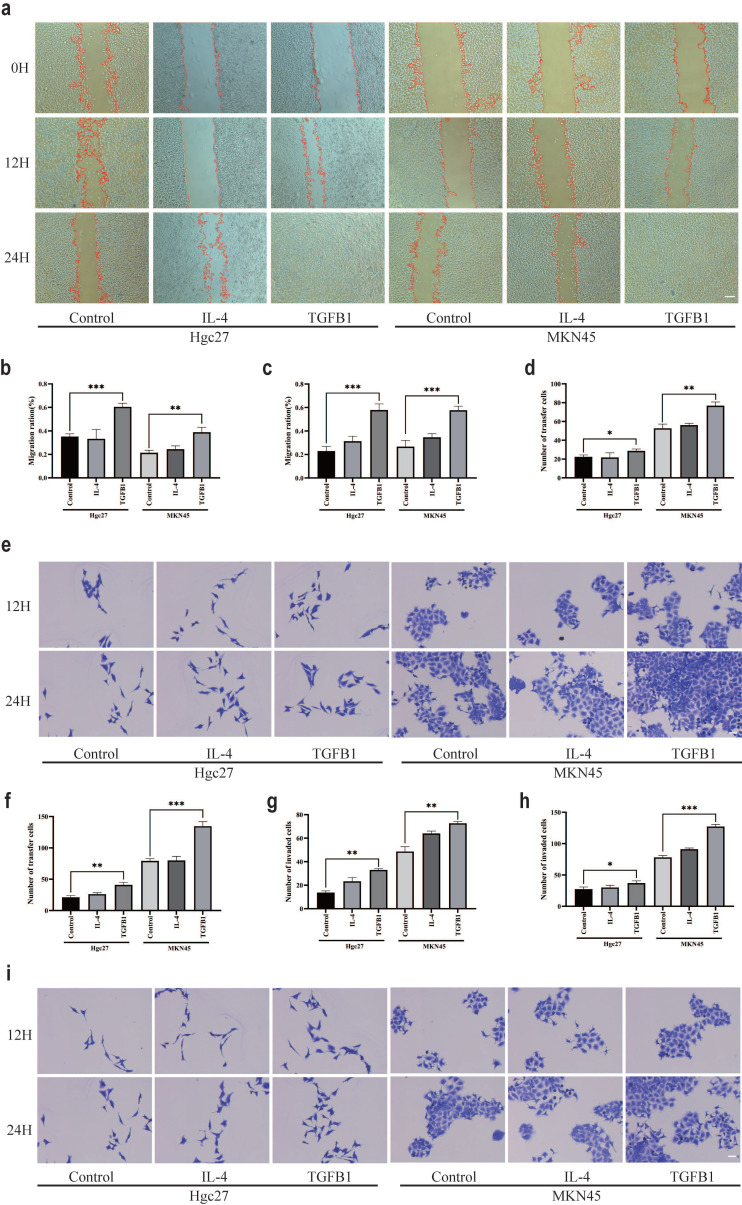
M2 subtype macrophages have an impact on the function of gastric cancer cells. **a** Scratch test results of gastric cancer cells (Hgc27 and MKN45) under different intervention conditions. **b** Statistical results of 12 h scratch test on gastric cancer cells (Hgc27 and MKN45). **c** Statistical results of 24 h scratch test on gastric cancer cells (Hgc27 and MKN45). **d** Statistical results of 12 h migration experiment of gastric cancer cells (Hgc27 and MKN45). **e** The migration experiment results of gastric cancer cells (Hgc27 and MKN45) under different intervention conditions. **f** Statistical results of 24 h migration experiment of gastric cancer cells (Hgc27 and MKN45). **g** Statistical results of 12 h invasion experiment of gastric cancer cells (Hgc27 and MKN45). **h** Statistical results of 24 h invasion experiment of gastric cancer cells (Hgc27 and MKN45). **i** The invasion experiment results of gastric cancer cells (Hgc27 and MKN45) under different intervention conditions. Scale bar=50 μm. *p<0.05, **p<0.01, ***p<0.001.

The effect of TGFβ1 on the prognosis of gastric adenocarcinoma is correlated with macrophage types, M2 subtype macrophages induced by different inducers play different roles in the intervention of gastric cancer cell lines. The scratch test results showed that the subtypes of macrophages formed by TGFβ1 intervention group were co cultured with gastric cancer cells (Hgc27 and MKN45) for 12 and 24 hours (Fig. 5a), and the proliferation ability of gastric cancer cells (Hgc27 and MKN45) was significantly enhanced (Fig. 5b-c). The results of the Transwell migration experiment showed that the migration results were not significant after 12 hours (Fig. 5d). After intervention with different subtypes of macrophage culture media for 24 hours, the number of migrating ventricular cells (Hgc27 and MKN45) in gastric cancer cells significantly increased, their migration ability was enhanced (Fig. 5e-f). The Transwell invasion experiment results showed that there was no significant invasion at 12 hours (Fig. 5g). After intervention with different subtypes of macrophage culture media for 12 and 24 hours, the number of lower ventricular gastric cancer cells (Hgc27 and MKN45) increased significantly, the invasion ability was enhanced (Fig. 5h-i).

### The effect of M2 subtype macrophages on TGFβ1-related pathway and epithelial mesenchymal transition in gastric cancer cells

**Figure 6:**
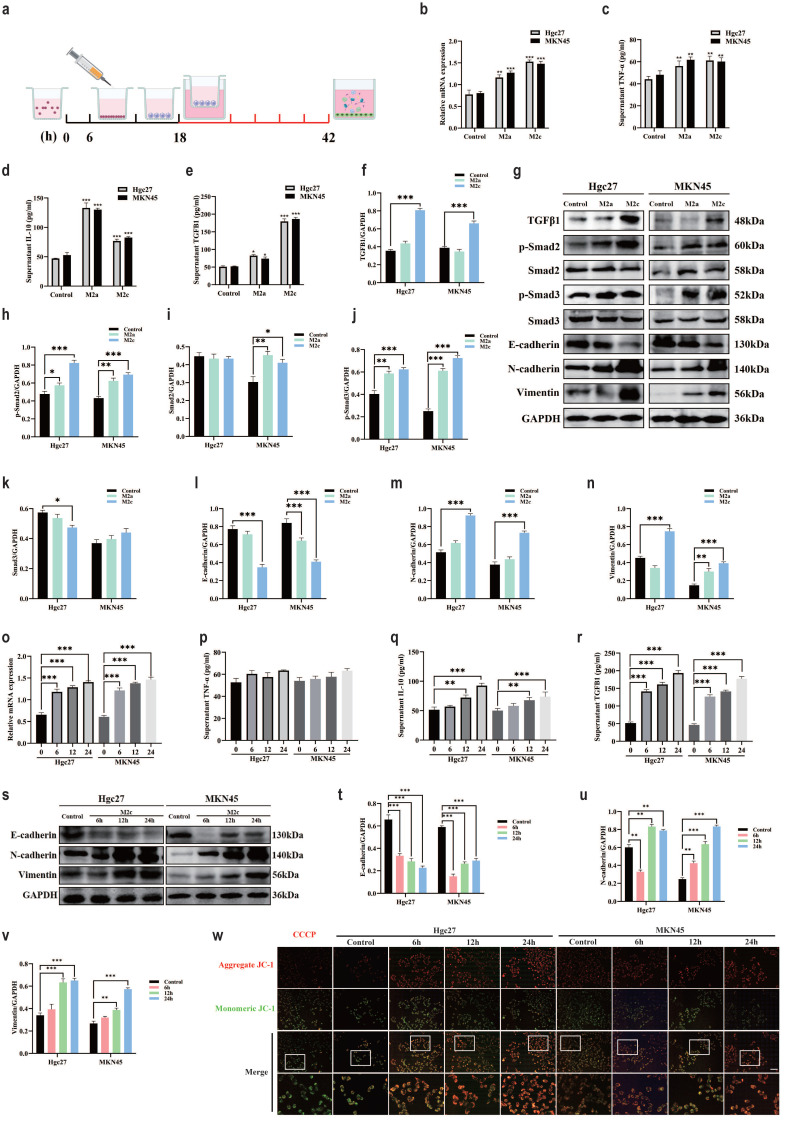
The effect of M2 subtype macrophages on TGFβ1 related pathway and epithelial mesenchymal transition in gastric cancer cells. **a** Cell intervention pattern diagram. **b** RT-qPCR was used to detect the levels of TGFβ mRNA in different intervention groups. **c** ELISA was used to test the expression of TNF-α in different intervention groups. **d** ELISA test the expression of IL-10 in different intervention groups. **e** ELISA was used to test the expression of TGFβ1 in different intervention groups. **f** The expression results of TGFβ1 protein. **g** The WB results of different interventions on histone expression. **h** The expression results of p-Smad2 protein. **i** The expression results of Smad2 protein. **j** The expression results of p-Smad3 protein. **k** The expression results of Smad3 protein. **l** The expression results of E-cadherin protein. **m** Results of N-cadherin protein expression. **n** Results of Vimentin protein expression. **o** RT-qPCR was used to detect the content of TGFβ mRNA at different intervention times. **p** ELISA was used to test the expression of TNF-α at different intervention times. **q** ELISA test the expression of IL-10 at different intervention times. **r** ELISA was used to test the expression of TGFβ1 at different intervention times. **s** WB results of protein expression at different intervention times. **t** The expression results of E-cadherin protein. **u** The expression results of N-cadherin protein. **v** Results of Vimentin protein expression. **w** Fluorescence results of mitochondrial membrane potential at different intervention times. Scale bar=50 μm. *p<0.05, **p<0.01, ***p<0.001.

Add THP-1 suspension to the upper chamber of Transwell chamber, add inducer after 6 h, co culture the upper chamber with a small dish containing gastric cancer cells in the lower chamber after 12 h. Continue co culturing for 24 h and extract gastric cancer cell proteins (Fig. 6a). RT-qPCR was used to verify the expression of TGFβ in gastric cancer cell lines, the results showed a significant increase in the M2c co culture group and a slight increase in the M2a co culture group (Fig. 6b). ELISA examined markers of macrophage polarization, TNF-α results showed a slight increase in the M2a co culture group and M2c co culture group (Fig. 6c). IL-10 results showed a significant increase in the M2a co culture group and a slight increase in the M2c co culture group (Fig. 6d). TGFβ1 results showed a significant increase in the M2c co culture group and a slight increase in the M2a co culture group (Fig. 6e). Changes in TGFβ1 affect the TGFβ family, which in turn affects the classic TGFβ1/Smad pathway in the body 46, 47. Existing research supports a close relationship between the TGFβ1/Smad pathway and epithelial mesenchymal transition, the occurrence of epithelial mesenchymal transition is closely related to cancer cell function 48, 49.

Therefore, we speculate that M2c macrophages have an impact on gastric cancer cell lines by altering TGFβ1, further activating the TGFβ1/Smad pathway, promoting epithelial mesenchymal transition of gastric cancer cells. To further validate our hypothesis, we conducted WB experiments to investigate the expression of TGFβ1/Smad pathway and key proteins involved in epithelial mesenchymal transition (Fig. 6g). The WB results showed that compared with the blank group, the M2c co culture group had high expression of TGFβ1 protein in gastric cancer cells (Hgc27 and MKN45) (Fig. 6f). Compared with the blank group, the expression of p-Smad2 protein in gastric cancer cells (Hgc27 and MKN45) in the M2c co culture group showed the same trend (Fig. 6h), while there was a significant difference in Smad2 protein expression between the two gastric cancer cell lines in the M2c co culture group. The MKN45 cell group showed a significant difference, while Hgc27 showed no significant change (Fig. 6i). Compared with the blank group, the expression of p-Smad3 protein in gastric cancer cells (Hgc27 and MKN45) in the M2c co culture group showed the same trend (Fig. 6j), while there was a significant difference in Smad3 protein expression between the two gastric cancer cell lines M2c co culture groups. The Hgc27 cell group showed a significant difference, while MKN45 showed no significant change (Fig. 6k). Compared with the blank group, the co culture group of two gastric cancer cell lines M2c showed a significant decrease in E-cadherin protein (Fig. 6l), a significant increase in N-cadherin protein (Fig. 6m), a significant increase in Vimentin protein (Fig. 6n). To further investigate the effect of M2c co culture on epithelial mesenchymal transition in gastric cancer cell lines, we compared the expression of key proteins involved in epithelial mesenchymal transition in gastric cancer cell lines at different time periods. The RT-qPCR validation of TGFβ1 mRNA expression in gastric cancer cell lines showed that gastric cancer cells (Hgc27 and MKN45) showed varying degrees of elevation after 6 h, 12 h and 24 h of M2c intervention, this change was positively correlated with the intervention time (Fig. 6o). ELISA examined the markers of macrophage polarization, the TNF-α results showed no significant changes in the M2c co culture group at four time periods (Fig. 6p). IL-10 results showed a slight increase in the M2c co culture group after intervention for 12 h and 24 h (Fig. 6q). TGFβ1 results showed a significant increase in the M2c co culture group after intervention for 6 h, 12 h and 24 h (Fig. 6r). The WB results showed (Fig. 6s) that compared with the blank group, the E-cadherin protein significantly decreased (Fig. 6t), the N-cadherin protein significantly increased (Fig. 6u) in the M2c co culture group at three different time periods. Vimentin protein significantly increased at the intervention time of 12 h and 24 h (Fig. 6v). Mitochondrial membrane potential fluorescence detection was used to clarify the effect of M2c co culture on mitochondrial function in gastric cancer cells (Hgc27 and MKN45). The JC-1 fluorescence results showed that after co culturing M2c for 6 h, 12 h and 24 h, gastric cancer cells (Hgc27 and MKN45) were mainly composed of JC-1 polymer (red light) (Fig. 6w), mitochondrial function was not significantly affected.

### M2c macrophages increase ferroptosis resistance in gastric cancer cells

**Figure 7:**
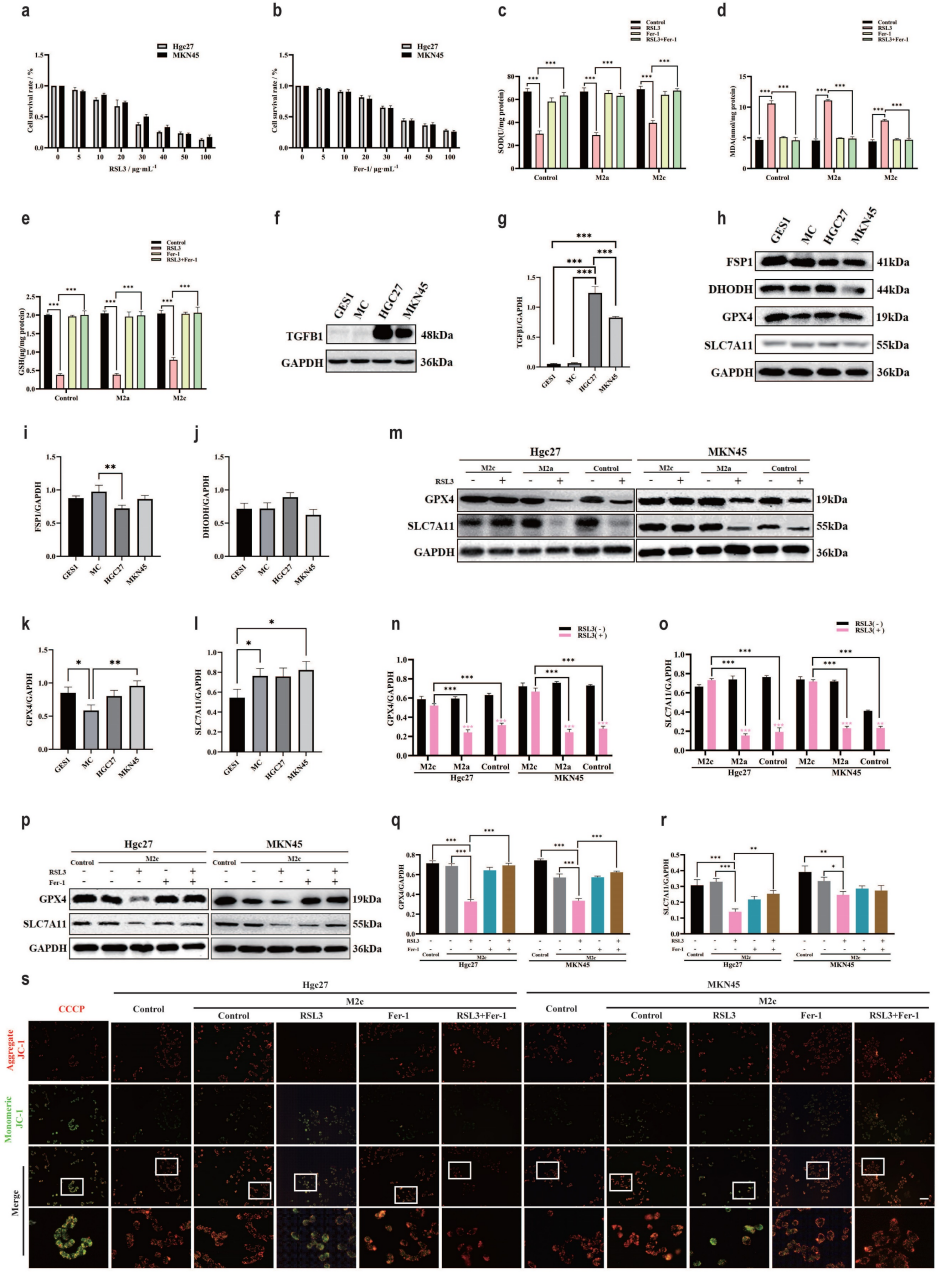
M2c macrophages increase ferroptosis resistance in gastric cancer cells. **a** The CCK-8 method was used to detect the survival of gastric cancer cells (Hgc27 and MKN45) intervened with RSL3 for 24 h. **b** The CCK-8 method was used to detect the survival of gastric cancer cells (Hgc27 and MKN45) intervened with Fer-1 for 24 h. **c** The expression of SOD in different intervention groups. **d** The expression of MDA in different intervention groups. **e** The expression of GSH in different intervention groups. **f** The expression of TGFβ1 protein WB in different cell lines. **g** The expression results of TGFβ1 protein. **h** The expression of key ferroptosis proteins WB in different cell lines. **i** The expression results of FSP1 protein. **j** Expression results of DHODH protein. **k** Expression results of GPX4 protein. **l** SLC7A11 protein expression results. **m** The intervention of RSL3 on the expression of key ferroptosis protein WB in different co culture groups. **n** The expression results of GPX4 protein. **o** SLC7A11 protein expression results. **p** The WB expression of key proteins involved in ferroptosis in different intervention groups. **q** The expression results of GPX4 protein. **r** The expression results of SLC7A11 protein. **s** Fluorescence results of mitochondrial membrane potential in different intervention groups. Scale bar=50 μm. *p<0.05, **p<0.01, ***p<0.001.

Ferroptosis is a classic non programmed mode of death that is influenced by specific environmental conditions 50. To clarify the intervention effect of macrophage subtypes on ferroptosis in gastric cancer cells in the TME, we used the classic inducer of ferroptosis RSL3 to reduce GPX4, Fer-1 antagonized RSL3 to inhibit ferroptosis 51, 52. The CCK-8 method was used to detect the effects of RSL3 and Fer-1 on the survival rate of gastric cancer cells (Hgc27 and MKN45). The results showed that the optimal intervention concentration of RSL3 was 20 μg·mL^-1^ (Fig. 7a), and the optimal intervention concentration of Fer-1 was 30 μg·mL^-1^ (Fig. 7b). SOD, MDA and GSH are classic indicators for measuring oxidation-reduction. Compared with the blank group, the small molecule compound RSL3 in the M2c group significantly inhibited the weakening of SOD and GSH (Fig. 7c, 7e), the enhancement of MDA was also inhibited (Fig. 7d). The results of SOD, MDA and GSH suggest that the metabolites of M2c macrophages seem to antagonize the occurrence of ferroptosis in gastric cancer cell lines. To further clarify the association between ferroptosis and M2c macrophages in the TME, we confirmed through WB results that the expression of TGFβ1 protein in gastric cancer cells (Hgc27 and MKN45) was higher than that in normal gastric mucosal cell line GES1 and atrophic gastritis model cell line MC (Fig. 7f-g). The WB results of ferroptosis key protein (Fig. 7h) showed that compared with GES1 and MC, there were no significant changes in FSP1 and DHODH in gastric cancer cells (Hgc27 and MKN45) (Fig. 7i-j), slight changes in GPX4 (Fig. 7k), no significant changes in SLC7A11 (Fig. 7l). Therefore, we speculate that GPX4 and downstream SLC7A11 are important ferroptosis intervention proteins in gastric cancer cells (Hgc27 and MKN45).

WB results showed (Fig. 7m) that compared with the blank group added with RSL3, the M2c co culture group added with RSL3 gastric cancer cells (Hgc27 and MKN45) showed no significant decrease in GPX4 protein (Fig. 7n) and downstream SLC7A11 protein (Fig. 7o). To further confirm that the transformation of GPX4 and SLC7A11 in the M2c co culture group was influenced by RSL3, we added the RSL3 antagonist Fer-1 to the co culture group. WB results showed that compared with the blank group, RSL3 promoted a decrease in GPX4 and SLC7A11 protein expression, which was inhibited by Fer-1 (Fig. 7p-r). Similarly, mitochondrial membrane potential fluorescence results showed that in gastric cancer cells (Hgc27 and MKN45), the RSL3 intervention group had an increase in JC-1 monomers (green light), Fer-1 inhibited the changes in mitochondria caused by RSL3 (Fig. 7s).

### The effect of gastric cancer cells on macrophage remodeling in the TME

**Figure 8:**
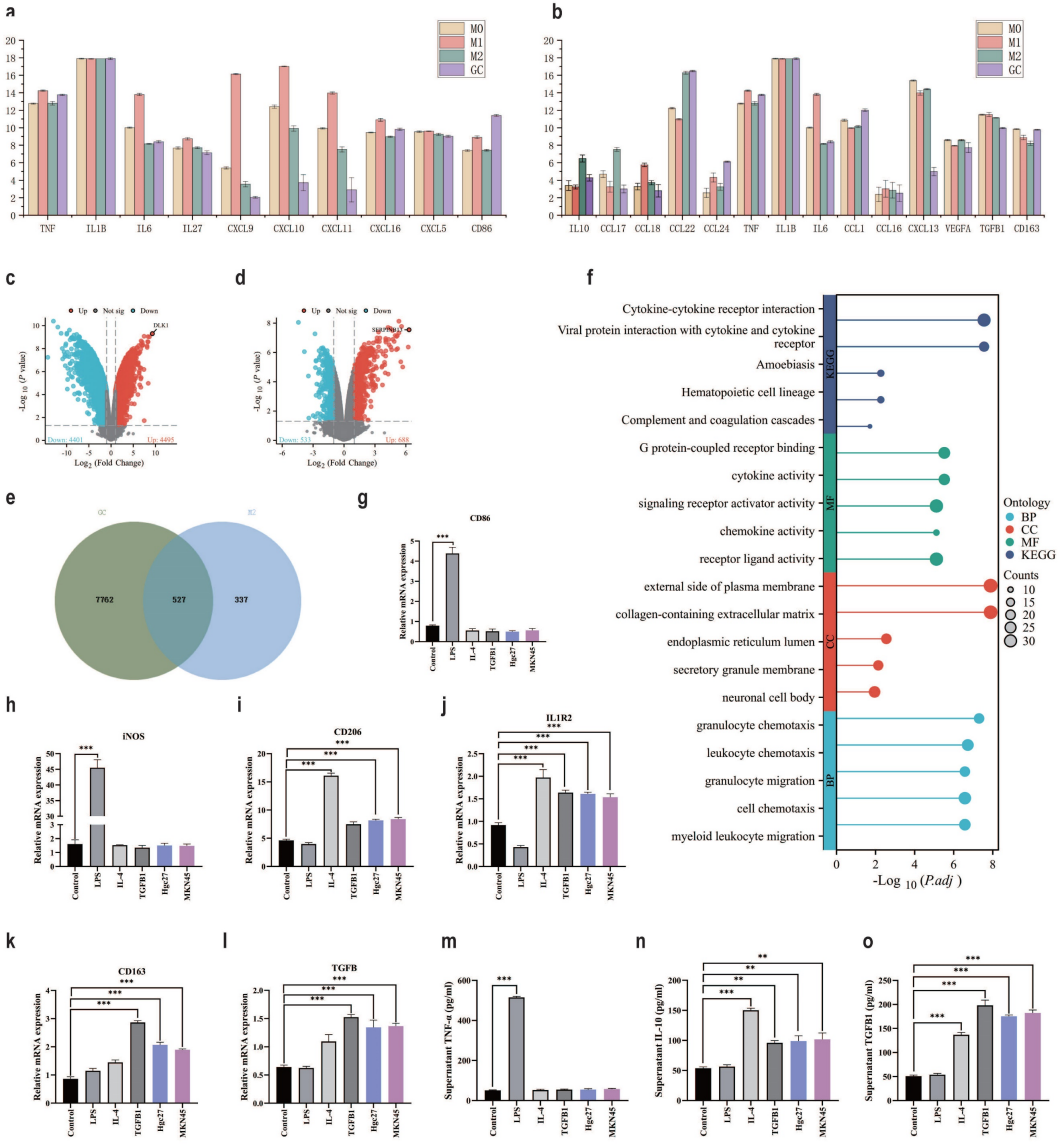
The role of gastric cancer cells in transforming macrophages in the TME. **a** The expression of M1 macrophage marker proteins in different groups. **b** The expression of M2 macrophage marker proteins in different groups. **c** Differential gene expression between M0 and gastric cancer cell metabolite intervention group. **d** Differential gene expression between M2 and gastric cancer cell metabolite intervention group. **e** Intersection statistics of differentially expressed genes between M2 and gastric cancer cell metabolite intervention group. **f** Intersection gene enrichment statistics. **g** RT-qPCR was used to detect the expression of CD86 mRNA. **h** RT-qPCR was used to detect the expression of iNOS mRNA. **i** RT-qPCR was used to detect the expression of CD206 mRNA. **j** RT-qPCR was used to detect the expression of IL1R2 mRNA. **k** RT-qPCR was used to detect the expression of CD163 mRNA. **l** RT-qPCR was used to detect the expression of TGFβ mRNA. **m** ELISA was used to detect TNF-α levels. **n** ELISA detects IL-10 levels. **o** ELISA detects the content of TGFβ1. *p<0.05; **p<0.01; ***p<0.001.

The TME is a complex microecological landscape, with complex cell types and transmission factors promoting further cancer progression 53. Immune escape is an important function of the TME, directly affecting cancer treatment 54. Focusing on the relationship between cancer cells and immune cells is of great significance for understanding immune escape 55. GSE146895 is a dataset in the GEO database that focuses on the effect of gastric cancer cell metabolites on macrophage polarization. We will compare the expression of macrophage polarization markers protein under the intervention conditions of gastric cancer cell metabolites, M1 and M2 in the dataset. In the comparison of metabolite interference group and M1 group marker proteins in gastric cancer cells, there were significant differences in IL-6, CXCL9, CXCL10 and CXCL11 (Fig. 8a). In the comparison of metabolite interference group and M2 group marker proteins in gastric cancer cells, there were significant differences in CCL17, CCL18 and CXCL13 (Fig. 8b). Therefore, we speculate that metabolites from gastric cancer cells may promote macrophage polarization towards M2, but the specific M2 subtype deserves further investigation. Comparing the metabolites of gastric cancer cells and the differentially expressed genes under M0 and M2 intervention conditions, it was found that compared with the M0 group, the metabolite interference group of gastric cancer cells significantly promoted the appearance of DLK1 in macrophages (Fig. 8c). Compared with the M2 group, the metabolic interference group of gastric cancer cells led to significant differences in SERPINB13 expression in macrophages (Fig. 8d). To further clarify the interference of gastric cancer cell metabolites on cellular processes, the intersection of differential genes between the M2 group and the cancer cell metabolite interference group was taken, a total of 527 intersection genes were obtained (Fig. 8e). These intersection genes may be important genes for gastric cancer cell metabolites to interfere with macrophage M2 polarization. Enrichment analysis of intersecting genes showed that BP enrichment mainly focused on macrophage chemotaxis and migration, CC enrichment mainly focused on extracellular matrix and external side of plasma membrane, MF enrichment mainly focused on cytokine activity and receptor ligand activity. The KEGG enrichment results showed that viral protein interaction with cytokines and cytokine receptors, as well as cytokine cytokine receptor interaction (Fig. 8f). RT-qPCR further clarified the effect of gastric cancer cell metabolites on macrophage polarization. The results of CD86 mRNA and iNOS mRNA showed no significant changes in the intervention group of gastric cancer cells (Hgc27 and MKN45) (Fig. 8g-h), CD206 mRNA and IL1R2 mRNA showed an increase in the intervention group of gastric cancer cells (Hgc27 and MKN45) (Fig. 8i-j), CD163 mRNA and TGFβ mRNA showed a significant increase in the intervention group of gastric cancer cells (Hgc27 and MKN45) (Fig. 8k-l), ELISA examined markers of macrophage polarization, TNF-α results showed no significant changes in the intervention group of gastric cancer cells (Hgc27 and MKN45) (Fig. 8m), IL-10 The results showed a slight increase in gastric cancer cells (Hgc27 and MKN45) intervention group (Fig. 8n), while TGFβ1 showed a significant increase in gastric cancer cells (Hgc27 and MKN45) intervention group (Fig. 8o).

## Discussion

GC is one of the most common malignant tumors worldwide, with fast metastasis and high mortality rate 56. The traditional treatment plan cannot effectively improve the prognosis of GC patients, so there is an urgent need for new ideas to break the deadlock 57, 58. This study focuses on the correlation between macrophages and cancer cells in TME, exploring the mechanism of action of different subtypes of M2 macrophages on gastric adenocarcinoma.

TGFβ1 is a familiar cell secreted factor, mainly present in extracellular matrix 59, 60. TGFβ1 has shown potential research value in the study of gastric adenocarcinoma and TME 61-64. The results of a large dataset from TCGA and GEO confirm that the content of TGFβ1 in gastric adenocarcinoma is higher than that in normal gastric tissue. Further analysis of clinical features revealed a negative correlation between the expression of TGFβ1 and the survival time of gastric adenocarcinoma patients, and a high correlation between TGFβ1 and T staging. Further analysis of immune infiltration in TME revealed a high correlation between macrophage marker proteins and TGFβ1 in gastric adenocarcinoma, with multiple pieces of evidence suggesting that macrophages are more intervened by TGFβ1 65, 66. There is no significant correlation between the expression of some macrophage marker proteins and TGFβ1, indicating that macrophage subtypes and polarization are the focus of TGFβ1 research in gastric adenocarcinoma. In TME, M1 and M2 macrophages are functionally antagonistic to each other, TGFβ1 is involved in the polarization of M2 macrophages 67, 68. In GC samples, there were significant differences in the expression of specific cytokines related to different subtypes of macrophages (M1, M2a, M2b, M2c and M2d). All M2 subtype macrophage markers were altered, with M2c macrophage markers showing significant changes. Both the highly metastatic gastric cancer dataset and the Helicobacter pylori infection dataset exhibit similar trends in TGFβ1 and M2c macrophages, indicating a close association between TGFβ1 and M2c in gastric adenocarcinoma. We have confirmed through extensive experiments examining macrophage polarization and subtypes that LPS promotes M1 polarization, IL-4 promotes M2a polarization, TGFβ1 promotes M2c polarization.

To further investigate the effects of different subtypes of macrophages on the function of gastric cancer cells, a cell co culture system was constructed using Transwell chambers. Cell scratch test, migration test, invasion test showed that after co culturing M2c macrophages induced by TGFβ1, the functions of two types of gastric cancer cells (Hgc27 and MKN45) were enhanced to varying degrees. This suggests the possibility of interaction between M2c macrophages and gastric cancer cells in TME, which can enhance the migration and invasion ability of gastric cancer cells. TGFβ1 is an important member of the TGFβ family and can activate the classical TGFβ1/Smad pathway 69. The abnormal regulation of the TGFβ1/Smad pathway plays a crucial role in the pathogenesis of many cancers, including gastric adenocarcinoma 70. Changes in the TGFβ1/Smad pathway can affect the progression of epithelial mesenchymal transition. Therefore, we speculate that the enhanced migration and invasion ability of gastric cancer cells in the co culture group of M2c macrophages induced by TGFβ1 is related to the activation of the TGFβ1/Smad pathway. WB experiments confirmed that the co culture group of M2c macrophages induced by TGFβ1 intervened in the expression of TGFβ1, Smad2 and Smad3 proteins, activated the TGFβ1/Smad pathway, further affected the process of epithelial mesenchymal transition. A series of co culture time point experiments further confirmed that the changes in epithelial mesenchymal transition of gastric cancer cells were caused by M2c co culture, the mitochondrial function of gastric cancer cells was not affected.

Ferroptosis is a non programmed death that is influenced by the environment 71. Ferroptosis reflects the accumulation of iron dependent lipid peroxides 72. Some studies suggest that TGFβ1 promotes ferroptosis and inhibits tumor growth 73. However, our extensive research and data in gastric adenocarcinoma do not support the view that TGFβ1 inhibits tumor growth 74-76. TGFβ1 is involved in the polarization process of M2c macrophages, promoting the occurrence of immune escape. High expression of TGFβ1 is negatively correlated with patient survival prognosis. Therefore, we speculate that TGFβ1 promoted M2c macrophage metabolites are closely associated with ferroptosis resistance in gastric adenocarcinoma TME. After adding RSL3 to the M2c macrophage co culture group induced by TGFβ1 77, there was no significant decrease in SOD and GSH levels, nor a significant increase in MDA levels in gastric cancer cells. Comparing the expression of key proteins involved in ferroptosis in different cells, GPX4 and downstream SLC7A11 may be valuable evaluation indicators for gastric cancer cells (Hgc27 and MKN45). Similarly, after adding RSL3 to the TGFβ1-induced M2c macrophage co culture group, the decrease in GPX4 and SLC7A11 in gastric cancer cells was inhibited 51, 78. The mitochondrial membrane potential showed that the fluorescence intensity of mitochondrial JC-1 monomer increased after the addition of RSL3. And the Fer-1 antagonism results showed that M2c enhances ferroptosis resistance in gastric cancer cells (Hgc27 and MKN45).

Existing research has confirmed the effect of gastric cancer cell metabolites on macrophage polarization 79, 80. These studies focus on the polarization bias of gastric cancer cell metabolites towards macrophages, namely pro-inflammatory M1 and anti-inflammatory M2 81, 82. Obviously, gastric cancer metabolites can promote more M0 macrophages to polarize towards M2 type, but further research on M2 macrophage subtypes has not been conducted 83. In TME, different subtypes of M2 macrophages participate in different processes and contribute significantly to immune escape 84, 85. Metabolites from gastric cancer cells activate a large number of genes in macrophages, most of which are related to M2 macrophages. The enrichment results of these genes indicate that the macrophage polarization pathway is activated. Using two metabolites from gastric cancer cells (Hgc27 and MKN45) to affect M0 macrophages, in M1 marker detection, the metabolites from gastric cancer cells (Hgc27 and MKN45) showed high similarity with IL-4 and TGFβ1, confirming the correctness of the conclusion that gastric cancer cell metabolites can promote more M0 macrophages to polarize towards M2 type. However, in the M2 subtype detection, our research results only support that the metabolites of gastric cancer cells (Hgc27 and MKN45) have a similar effect on M2 subtype macrophages to TGFβ1. It cannot be ruled out that some M2a macrophages may be produced, which is related to the interconversion between M2 macrophage subtypes. More researchers have observed through different experiments that there is a transformation relationship between M2 macrophage subtypes, but the relevant mechanism is not yet clear. More complex experiments may elucidate the transformation relationship between M2 macrophage subtypes. But our research has clearly elucidated the mutually beneficial symbiotic relationship between M2c macrophages and gastric cancer cells. M2c macrophages contribute a stronger protective mechanism to gastric cancer cells, preventing the occurrence of ferroptosis and metastasis inhibition. Gastric cancer cells contribute reliable polarization influencing factors to M2c macrophages, promoting their production.

## Conclusion

In summary, our study reveals a mutually beneficial symbiotic relationship between M2c macrophages and cancer cells in the microenvironment of gastric cancer tumors. TGFβ1 promotes the production of M2c macrophages, which enhance the function and ferroptosis resistance of gastric cancer cells. Gastric cancer cells provide the material basis for M2c macrophage polarization. This new evidence may provide new insights into developing more effective targeted therapies for gastric cancer to combat the formation of immune escape and metastasis in gastric cancer.

## Supplementary Material

Supplementary tables.

## Abbreviations

GC: Gastric cancer
TME: Tumor microenvironment
DEGs: Differentially expressed genes
DMEM: Dulbecco's modified Eagle's medium
FBS: Fetal bovine serum
CD86: Cluster of differentiation 86
iNOS: Inducible nitric oxide synthase
CD206: Cluster of differentiation 206
IL1R2: Interleukin-1 receptor type 2
CD163: Cluster of differentiation 163
TGFB: Transforming growth factor β
TGFβ: Transforming growth factor β
GAPDH: Glyceraldehyde 3 phosphate dehydrogenase
GPX4: Phospholipid hydroperoxide glutathione peroxidase
SLC7A11: Cystine/glutamate transporter
FSP1: Ferroptosis suppressor protein 1
DHODH: Dihydroorotate dehydrogenase (quinone)
TGFB1: Transforming growth factor β 1
TGFβ1: Transforming growth factor β 1
Smad2: Mothers against decapentaplegic homolog 2
p-Smad2: phospho-mothers against decapentaplegic homolog 2
Smad3: Mothers against decapentaplegic homolog 3
p-Smad3: phospho-mothers against decapentaplegic homolog 3
E-cadherin: Cadherin-1
N-cadherin: Cadherin-2
RSL3: (1S,3R)-RSL3
Fer-1: Ferrostatin-1
MDA: Malondialdehyde
SOD: Superoxide dismutase
GSH: Reduced glutathione
RT‒qPCR: Real-time quantitative polymerase chain reaction
