# Supplementary material for: The mutualistic relationship between M2c macrophages of TGFβ1 induction and gastric cancer cells: the correlation between protective mechanisms in the tumor microenvironment and polarization of subtypes of cells
Source: J Cancer. 2025 Feb 3;16(5):1598–617. doi: 10.7150/jca.97784 (PMC11843238; doi:10.7150/jca.97784)
Supplement: Supplementary file 1 — Supplementary tables. [file jcav16p1598s1.pdf]

Table S1

GEO digital.

Table S2

Summary of PCR Primers

Table S3

TCGA - STAD digital.

Table S4

Different macrophage subtype markers.

Table S1 GEO digital.

GEO dataset	Sample size	Characteristic	Describe	Quote
GSE146895	12	Integrated analysis of long non-coding RNAs expression profiles in THP-1 induced M0, M1 and M2 macrophage and GCC culture medium induced similar M2 (CM-sM2)	To screen for the functional lncRNAs involved in macrophages polarization, total RNA from THP-1 induced M0, M1, M2 and CM-sM2 cells were extracted and analyzed, we employed SBC-lncRNA (human 4*180K) microarray as a discovery platform to identify genes. [M0 were induced by PMA (50ng/ml) for 48h, M1 were induced by LPS (100ng/ml) and IFN-γ (20ng/ml) for 48h and M2 were induced by IL-4 (20ng/ml) for 48h both on the basis of M0. CM-sM2 were induced by 30% culture medium of gastric cancer cells on the	No citation
GSE65801	64	Characterization of differentially expressed genes involved in pathways associated with gastric cancer	To explore the patterns of gene expression in gastric cancer, a total of 32 paired gastric cancer and noncancerous tissues from patients were collected for gene expression microarray analyses. Limma methods were applied to analyze the data, and genes were considered to be significantly differentially expressed if the False Discovery Rate (FDR) values < 0.01, P-value < 0.01 and the fold change >2. Subsequently, Gene Ontology (GO) analysis was used to analyze the main functions of the differentially expressed genes. According to the Kyoto Encyclopedia of Genes and Genomes (KEGG) database, we found pathways significantly associated with the differential genes. Gene-Act network and Co-Expression networks were built respectively based on the relationships among the genes, proteins and compounds in the database. There were 2371 differential mRNAs and 350 differential lncRNAs in our microarray data. The GO categories, pathway analyses and the Gene-Act network showed a consistent result that up-regulated genes were involved in tumorigenesis, migration, angiogenesis and microenvironment formation, while down-regulated genes were involved in metabolism. The results of this study provide some novel findings on genes, [illegible]	[32]

GSE84787	20	Human gastric cancer_Tumor samples_Paired normal samples_10 replicates	To identify the lncRNA profiles of gastric cancer samples, we performed microarray analysis using tumor samples and paired normal samples. 66 lncRNAs were statistically dysregulated more than 2-fold.	[33]
GSE103236	19	Microarray gene expresion analysis for early and advanced gastric adenocarcinoma vs normal adjacent tissue.	The study aimed to understand gradual biological variations during gastric tumorigenesis, and to identify the candidate genes that are involved in tumor progression and metastasis. cDNA microarray data were obtained from 10 pair of cancerous and normal adjacent tissue from gastric adenocarcinoma patients. As a results, we found that 136 genes were up-regulated and 96 genes were down-regulated by at least four-fold in tumor tissue. The analysis of gene clusters revealed a complex remodelling of normal gastric epithelium morphology and function associated with the tumorigenesis and metastasis. A large number of proteases are being overexpressed, together with keratins, genes associated with morphogenesis and anti-apoptosis. Between the most significant down-regulated genes, were genes involved gastric motility and synthesis, genes related to metabolic and pro-apoptotic processes. We report also, the identification of seven genes, significant up-regulated, that seems to be associated with tumor progression: KRT17, COL10A1, KIAA1199, SPP1, IL11, S100A2, and MMP3. Conclusions. Our cDNA microarray study identified several genes that appeared to meet the criteria of a good biomarker, and may therefore be	[34]
GSE70394	6	Gene expression profiling of gastric cells infected by Helicobacter pylori	The human gastric carcinoma-derived cell line AGS was infected with H. pylori strain 60190 (ATCC 49503) for 24 hours. RNA was extracted from three independent	[35]

GSE21328	4	Transcriptome analysis of highly metastatic cell line	A prediction of peritoneal recurrence is of significance using metastasis-related biomarker. This work describes a combined analysis of proteome and transcriptome data for biomarker discovery in highly metastatic cell line. We used nano-flow liquid chromatography (LC) linear ion trap time-of-flight mass spectrometry (LIT-TOF MS) and cDNA microarray to identify specific protein differentially expressed between a highly metastatic stomach cancer cell line MKN-45-P and its parental cell line MKN-45. In total, 240 proteins were found to be expressed between the two cell lines. Of these, 75 proteins (31%) and 49 proteins (20%) were only identified from MKN-45-P and MKN-45 respectively. An mRNA expression of 1533 genes was up-regulated in MKN-45-P compared with MKN-45. No close correlation was found between proteomic and transcriptomic analysis. Interestingly, 4	[36]

Table S2 Summary of PCR Primers

Primer name	Target protein and abbreviation	sequences（5'-3'）
CD86	Cluster of differentiation 86	F:GAGCGGGATAGTAACGCTGA
		R:GGCTCTCACTGCCTTCACTC
iNOS	Inducible nitric oxide synthase (iNOS)	F:CCCTTCAATGGTTGGTACATGG
		R:ACATTGATCTCCGTGACAGCC
CD206	Cluster of differentiation 206	F:CTTCGGGCCTTTGGAATAAT
		R:TAGAAGAGCCCTTGGGTTGA
IL1R2	Interleukin-1 receptor type 2(IL1R2)	F:CCCTCAAGACCATATCAGCTTCTC
		R:TGTGCCGGTTCCCAGAAA
CD163	Cluster of differentiation 163	F:ACAATGAAGATGCTGGCGTGAC
		R:TCTCTGAATCTCCACCTCAACTGTC
TGFB	Transforming growth factor β (TGFβ)	F:CTTTTGACGTCACTGGAGTTG
		R:CAGTGAGCGCTGAATCGAA
GAPDH	Glyceraldehyde 3 phosphate	F:CAGGAGGCATTGCTGATGAT
		R:GAAGGCTGGGGCTCATTT

Table S3 TCGA - STAD digital.

Case ID	Project	Primary Site	Gender	Files	Seq	Exp	SNV	CNV	Meth	Clinical	Bio	Mutations	Genes	Slides	Program	Disease Type	Age at diagnosis	Days to death	Vital Status	Primary Diagnosis	Ethnicity	Race
TCGA-VQ-A8P2	TCGA-STAD	Stomach	Male	71	10	4	16	9	3	8	16	8088	5838	2	TCGA	Cystic, Mucinous and Serous Neoplasms	68 years 292 days	--	Alive	Mucinous adenocarcinoma	not reported	white
TCGA-BR-8680	TCGA-STAD	Stomach	Male	74	10	4	16	11	3	8	17	6617	4714	3	TCGA	Adenomas and Adenocarcinomas	45 years 201 days	--	Alive	Tubular adenocarcinoma	not hispanic or latino	asian
TCGA-BR-6452	TCGA-STAD	Stomach	Female	85	13	4	17	14	6	8	18	5014	4025	4	TCGA	Adenomas and Adenocarcinomas	78 years 338 days	--	Alive	Adenocarcinoma, NOS	not hispanic or latino	white

TCGA-CG-5721	TCGA-STAD	Stomach	Male	87	14	8	16	11	3	8	18	4865	3945	4	TCGA	Adenomas and Adenocarcinomas	58 years 60 days	--	Alive	Adenocarcinoma, intestinal type	not reported	not reported
TCGA-BR-4184	TCGA-STAD	Stomach	Male	67	7	2	17	7	6	8	19	3677	3055	5	TCGA	Adenomas and Adenocarcinomas	70 years 249 days	212 days	Dead	Adenocarcinoma, NOS	not hispanic or latino	white
TCGA-BR-8487	TCGA-STAD	Stomach	Female	70	10	4	16	9	3	8	16	3388	2870	2	TCGA	Adenomas and Adenocarcinomas	64 years 24 days	--	Alive	Adenocarcinoma, NOS	not hispanic or latino	white

TCGA-BR-4361	TCGA-STAD	Stomach	Female	77	8	4	16	11	6	8	19	2864	2457	5	TCGA	Adenomas and Adenocarcinomas	66 years 154 days	--	Alive	Adenocarcinoma, NOS	not hispanic or latino	white
TCGA-VQ-A91D	TCGA-STAD	Stomach	Male	73	10	4	16	11	3	8	16	2765	2409	2	TCGA	Adenomas and Adenocarcinomas	70 years 119 days	356 days	Dead	Adenocarcinoma, intestinal type	not reported	white
TCGA-CD-A4MG	TCGA-STAD	Stomach	Male	71	8	4	16	11	3	8	16	2703	2323	2	TCGA	Adenomas and Adenocarcinomas	76 years 260 days	200 days	Dead	Carcinoma, diffuse type	not hispanic or latino	asian

TCGA-HU-A4GQ	TCGA-STAD	Stomach	Male	74	9	6	16	11	3	8	16	2184	1964	2	TCGA	Adenomas and Adenocarcinomas	71 years 268 days	3 days	Dead	Tubular adenocarcinoma	not hispanic or latino	asian
TCGA-CG-4460	TCGA-STAD	Stomach	Female	75	10	4	16	11	3	9	17	2175	1918	3	TCGA	Adenomas and Adenocarcinomas	66 years 123 days	1 year 304 days	Dead	Adenocarcinoma, intestinal type	not reported	not reported
TCGA-BR-A4QL	TCGA-STAD	Stomach	Female	78	9	4	18	13	3	8	16	2114	1906	2	TCGA	Adenomas and Adenocarcinomas	75 years 275 days	1 year 126 days	Dead	Adenocarcinoma, NOS	not hispanic or latino	white

TCGA-CG-5723	TCGA-STAD	Stomach	Male	77	12	4	16	11	3	8	18	2063	1864	4	TCGA	Adenomas and Adenocarcinomas	83 years 61 days	--	Alive	Adenocarcinoma, NOS	not reported	not reported
TCGA-HU-8602	TCGA-STAD	Stomach	Female	75	9	4	17	12	3	8	17	1963	1736	3	TCGA	Adenomas and Adenocarcinomas	58 years 338 days	--	Alive	Carcinoma, diffuse type	not hispanic or latino	asian
TCGA-BR-8078	TCGA-STAD	Stomach	Female	70	8	4	16	11	3	8	16	1884	1720	2	TCGA	Adenomas and Adenocarcinomas	70 years 294 days	--	Alive	Adenocarcinoma, NOS	not hispanic or latino	white

TCGA-BR-8361	TCGA-STAD	Stomach	Female	72	8	4	16	11	3	8	17	1874	1676	3	TCGA	Adenomas and Adenocarcinomas	71 years 67 days	--	Alive	Adenocarcinoma, NOS	not hispanic or latino	white
TCGA-HU-A4G8	TCGA-STAD	Stomach	Female	70	8	4	16	11	3	8	16	1793	1661	2	TCGA	Adenomas and Adenocarcinomas	71 years 322 days	--	Alive	Carcinoma, diffuse type	not hispanic or latino	asian
TCGA-BR-4362	TCGA-STAD	Stomach	Female	68	5	2	16	11	6	8	19	1810	1640	5	TCGA	Adenomas and Adenocarcinomas	74 years 151 days	--	Alive	Adenocarcinoma, NOS	not hispanic or latino	white

TCGA-BR-8591	TCGA-STAD	Stomach	Male	74	10	4	16	11	3	8	17	1792	1635	3	TCGA	Adenomas and Adenocarcinomas	79 years 200 days	--	Alive	Adenocarcinoma, NOS	not hispanic or latino	white
TCGA-VQ-A8PP	TCGA-STAD	Stomach	Male	73	10	4	16	11	3	8	16	1719	1590	2	TCGA	Adenomas and Adenocarcinomas	76 years 15 days	1 year 347 days	Dead	Tubular adenocarcinoma	not reported	not reported
TCGA-FP-A4BE	TCGA-STAD	Stomach	Male	68	8	4	16	8	3	8	17	1731	1589	3	TCGA	Adenomas and Adenocarcinomas	55 years 60 days	--	Alive	Adenocarcinoma, NOS	not hispanic or latino	black or african american

TCGA-HU-A4GT	TCGA-STAD	Stomach	Female	71	8	4	16	11	3	8	16	1695	1568	2	TCGA	Adenomas and Adenocarcinomas	71 years 299 days	--	Alive	Tubular adenocarcinoma	not hispanic or latino	asian
TCGA-CG-4442	TCGA-STAD	Stomach	Male	76	12	4	16	11	3	8	17	1715	1552	3	TCGA	Adenomas and Adenocarcinomas	85 years 304 days	--	Alive	Adenocarcinoma, intestinal type	not reported	not reported
TCGA-MX-A5UJ	TCGA-STAD	Stomach	Female	72	10	4	16	11	3	8	16	1706	1550	2	TCGA	Adenomas and Adenocarcinomas	86 years 106 days	--	Alive	Tubular adenocarcinoma	not hispanic or latino	white

TCGA-BR-4201	TCGA-STAD	Stomach	Female	79	10	4	16	11	6	8	19	1701	1539	5	TCGA	Adenomas and Adenocarcinomas	66 years 248 days	2 years 210 days	Dead	Adenocarcinoma, NOS	not hispanic or latino	white
TCGA-HF-A5NB	TCGA-STAD	Stomach	Female	78	9	4	18	13	3	8	16	1653	1533	2	TCGA	Cystic, Mucinous and Serous Neoplasms	75 years 283 days	--	Alive	Mucinous adenocarcinoma	not reported	not reported
TCGA-HU-A4GU	TCGA-STAD	Stomach	Male	71	8	4	16	11	3	8	16	1695	1520	2	TCGA	Adenomas and Adenocarcinomas	73 years 361 days	--	Alive	Carcinoma, diffuse type	not hispanic or latino	asian

TCGA-CG-5728	TCGA-STAD	Stomach	Female	78	11	6	16	11	3	8	18	1646	1514	4	TCGA	Adenomas and Adenocarcinomas	88 years 151 days	--	Alive	Adenocarcinoma, intestinal type	not reported	not reported
TCGA-BR-7851	TCGA-STAD	Stomach	Male	90	13	8	17	14	3	8	18	1625	1498	4	TCGA	Adenomas and Adenocarcinomas	74 years 63 days	--	Dead	Adenocarcinoma, intestinal type	not hispanic or latino	white
TCGA-VQ-A8PT	TCGA-STAD	Stomach	Male	61	6	0	16	11	3	8	16	1619	1486	2	TCGA	Adenomas and Adenocarcinomas	65 years 56 days	--	Alive	Adenocarcinoma, intestinal type	not reported	white

TCGA-BR-4292	TCGA-STAD	Stomach	Female	78	10	4	16	11	6	8	18	1627	1482	4	TCGA	Adenomas and Adenocarcinomas	73 years 7 days	--	Alive	Adenocarcinoma, NOS	not hispanic or latino	white
TCGA-D7-A6EY	TCGA-STAD	Stomach	Female	73	10	4	16	11	3	8	16	1598	1469	2	TCGA	Adenomas and Adenocarcinomas	72 years 228 days	348 days	Dead	Tubular adenocarcinoma	not hispanic or latino	white
TCGA-VQ-A924	TCGA-STAD	Stomach	Male	72	10	4	16	11	3	8	16	1577	1450	2	TCGA	Adenomas and Adenocarcinomas	69 years 149 days	4 years 225 days	Dead	Tubular adenocarcinoma	not reported	not reported

TCGA-VQ-A91K	TCGA-STAD	Stomach	Male	73	10	4	16	11	3	8	16	1562	1439	2	TCGA	Adenomas and Adenocarcinomas	69 years 222 days	--	Alive	Adenocarcinoma, intestinal type	not reported	white
TCGA-F1-6177	TCGA-STAD	Stomach	Male	75	10	4	16	11	3	9	17	1534	1411	3	TCGA	Adenomas and Adenocarcinomas	90 years	--	Dead	Adenocarcinoma, NOS	not hispanic or latino	black or african american
TCGA-BR-8372	TCGA-STAD	Stomach	Male	72	8	4	16	11	3	8	17	1541	1398	3	TCGA	Adenomas and Adenocarcinomas	63 years 336 days	--	Alive	Adenocarcinoma, intestinal type	not hispanic or latino	white

TCGA-BR-4368	TCGA-STAD	Stomach	Female	79	10	4	16	11	6	8	19	1503	1388	5	TCGA	Adenomas and Adenocarcinomas	78 years 281 days	--	Alive	Adenocarcinoma, NOS	not hispanic or latino	white
TCGA-CG-5726	TCGA-STAD	Stomach	Male	75	10	4	16	11	3	8	18	1491	1386	4	TCGA	Adenomas and Adenocarcinomas	73 years 335 days	2 years 151 days	Dead	Adenocarcinoma, intestinal type	not reported	not reported
TCGA-BR-7707	TCGA-STAD	Stomach	Female	70	8	4	16	11	3	8	16	1488	1377	2	TCGA	Adenomas and Adenocarcinomas	69 years 123 days	--	Alive	Adenocarcinoma, NOS	not hispanic or latino	white

TCGA-HU-A4GX	TCGA-STAD	Stomach	Female	83	11	8	17	14	3	8	17	1479	1376	3	TCGA	Adenomas and Adenocarcinomas	70 years 346 days	--	Alive	Carcinoma, diffuse type	not hispanic or latino	asian
TCGA-BR-8363	TCGA-STAD	Stomach	Female	74	10	4	16	11	3	8	17	1467	1361	3	TCGA	Adenomas and Adenocarcinomas	73 years 313 days	8 days	Dead	Adenocarcinoma, NOS	not hispanic or latino	white
TCGA-CG-4305	TCGA-STAD	Stomach	Male	74	10	4	16	11	3	8	17	1454	1336	3	TCGA	Adenomas and Adenocarcinomas	69 years 93 days	--	Alive	Adenocarcinoma, NOS	not reported	not reported

TCGA-HF-7132	TCGA-STAD	Stomach	Male	72	8	4	16	11	3	8	17	1440	1323	3	TCGA	Cystic, Mucinous and Serous Neoplasms	--	--	Alive	Mucinous adenocarcinoma	not reported	not reported
TCGA-HU-A4H8	TCGA-STAD	Stomach	Male	70	8	4	16	11	3	8	16	1395	1281	2	TCGA	Adenomas and Adenocarcinomas	77 years 168 days	--	Not Reported	Tubular adenocarcinoma	not hispanic or latino	asian
TCGA-CG-4306	TCGA-STAD	Stomach	Male	74	10	4	16	11	3	8	17	1312	1234	3	TCGA	Adenomas and Adenocarcinomas	89 years 364 days	1 day	Dead	Adenocarcinoma, NOS	not reported	not reported

TCGA-BR-4257	TCGA-STAD	Stomach	Female	79	10	4	16	11	6	8	19	1315	1225	5	TCGA	Adenomas and Adenocarcinomas	79 years 160 days	294 days	Dead	Adenocarcinoma, NOS	not hispanic or latino	white
TCGA-B7-5816	TCGA-STAD	Stomach	Female	73	10	4	16	11	3	8	16	1297	1213	2	TCGA	Adenomas and Adenocarcinomas	51 years 71 days	--	Alive	Carcinoma, diffuse type	not hispanic or latino	white
TCGA-HU-A4GN	TCGA-STAD	Stomach	Male	86	13	8	17	12	3	8	17	1303	1203	3	TCGA	Adenomas and Adenocarcinomas	61 years 8 days	--	Alive	Tubular adenocarcinoma	not hispanic or latino	asian

TCGA-CG-5733	TCGA-STAD	Stomach	Female	78	11	6	16	11	3	8	18	1306	1201	4	TCGA	Adenomas and Adenocarcinomas	83 years 335 days	1 year 276 days	Dead	Adenocarcinoma, intestinal type	not reported	not reported
TCGA-BR-4370	TCGA-STAD	Stomach	Female	79	10	4	16	11	6	8	19	1279	1187	5	TCGA	Adenomas and Adenocarcinomas	74 years 316 days	--	Alive	Adenocarcinoma, NOS	not hispanic or latino	white
TCGA-BR-6852	TCGA-STAD	Stomach	Female	94	17	8	17	14	3	8	18	1229	1133	4	TCGA	Adenomas and Adenocarcinomas	64 years 93 days	--	Alive	Adenocarcinoma, NOS	not hispanic or latino	white

TCGA-CD-A4MI	TCGA-STAD	Stomach	Male	69	8	4	16	9	3	8	16	1190	1116	2	TCGA	Adenomas and Adenocarcinomas	62 years 76 days	358 days	Dead	Adenocarcinoma, NOS	not hispanic or latino	asian
TCGA-BR-8368	TCGA-STAD	Stomach	Female	72	8	4	16	11	3	8	17	1173	1102	3	TCGA	Adenomas and Adenocarcinomas	84 years 193 days	--	Alive	Adenocarcinoma, NOS	not hispanic or latino	white
TCGA-BR-4256	TCGA-STAD	Stomach	Male	77	8	4	16	11	6	8	19	1160	1093	5	TCGA	Adenomas and Adenocarcinomas	80 years 221 days	284 days	Dead	Adenocarcinoma, NOS	not hispanic or latino	white

TCGA-BR-8589	TCGA-STAD	Stomach	Male	72	8	4	16	11	3	8	17	1187	1081	3	TCGA	Adenomas and Adenocarcinomas	56 years 273 days	--	Alive	Adenocarcinoma, NOS	not hispanic or latino	white
TCGA-HU-A4H3	TCGA-STAD	Stomach	Female	66	6	4	16	9	3	8	15	1110	1036	1	TCGA	Adenomas and Adenocarcinomas	56 years 336 days	--	Alive	Carcinoma, diffuse type	not hispanic or latino	asian
TCGA-BR-8382	TCGA-STAD	Stomach	Female	72	8	4	16	11	3	8	17	1088	1014	3	TCGA	Adenomas and Adenocarcinomas	67 years 266 days	2 years 32 days	Dead	Adenocarcinoma, NOS	not hispanic or latino	white

TCGA-BR-8081	TCGA-STAD	Stomach	Female	72	8	4	16	11	3	8	17	1068	1008	3	TCGA	Adenomas and Adenocarcinomas	71 years 163 days	--	Alive	Adenocarcinoma, NOS	not hispanic or latino	white
TCGA-D7-A4YV	TCGA-STAD	Stomach	Female	71	8	4	16	11	3	8	16	1054	1006	2	TCGA	Adenomas and Adenocarcinomas	69 years 219 days	180 days	Dead	Tubular adenocarcinoma	not hispanic or latino	white
TCGA-F1-6874	TCGA-STAD	Stomach	Male	76	12	4	16	11	3	8	17	1070	1001	3	TCGA	Adenomas and Adenocarcinomas	79 years 229 days	--	Alive	Adenocarcinoma, intestinal type	not hispanic or latino	white

TCGA-CD-8536	TCGA-STAD	Stomach	Male	72	8	4	16	11	3	8	17	1044	986	3	TCGA	Adenomas and Adenocarcinomas	74 years 300 days	--	Alive	Adenocarcinoma, NOS	not hispanic or latino	asian
TCGA-BR-8360	TCGA-STAD	Stomach	Male	63	5	2	16	11	3	8	17	1047	983	3	TCGA	Adenomas and Adenocarcinomas	66 years 288 days	--	Alive	Adenocarcinoma, intestinal type	not hispanic or latino	white
TCGA-VQ-A8PO	TCGA-STAD	Stomach	Male	73	10	4	16	11	3	8	16	1009	959	2	TCGA	Cystic, Mucinous and Serous Neoplasms	74 years 222 days	282 days	Dead	Signet ring cell carcinoma	not reported	white

TCGA-BR-6566	TCGA-STAD	Stomach	Female	83	11	4	17	14	6	8	18	1008	950	4	TCGA	Adenomas and Adenocarcinomas	64 years 28 days	--	Alive	Adenocarcinoma, NOS	not hispanic or latino	white
TCGA-R5-A7ZI	TCGA-STAD	Stomach	Female	71	10	4	16	11	3	8	15	1001	940	1	TCGA	Adenomas and Adenocarcinomas	44 years 325 days	--	Alive	Carcinoma, diffuse type	hispanic or latino	white
TCGA-VQ-A8PB	TCGA-STAD	Stomach	Female	72	10	4	16	11	3	8	16	943	894	2	TCGA	Adenomas and Adenocarcinomas	65 years 186 days	2 years 313 days	Dead	Papillary adenocarcinoma, NOS	not reported	white

TCGA-HU-A4G9	TCGA-STAD	Stomach	Female	84	13	8	17	14	3	8	17	921	870	3	TCGA	Adenomas and Adenocarcinomas	67 years 282 days	--	Alive	Tubular adenocarcinoma	not hispanic or latino	asian
TCGA-HJ-7597	TCGA-STAD	Stomach	Female	73	10	4	16	11	3	8	16	889	849	2	TCGA	Cystic, Mucinous and Serous Neoplasms	71 years 332 days	2 years 75 days	Dead	Signet ring cell carcinoma	not hispanic or latino	white
TCGA-VQ-A91E	TCGA-STAD	Stomach	Female	77	9	4	18	13	3	8	16	892	842	2	TCGA	Adenomas and Adenocarcinomas	67 years 78 days	--	Alive	Adenocarcinoma, intestinal type	not reported	white

TCGA-VQ-A8E3	TCGA-STAD	Stomach	Male	71	8	4	16	11	3	8	16	867	823	2	TCGA	Adenomas and Adenocarcinomas	79 years 65 days	1 year 296 days	Dead	Adenocarcinoma, NOS	not reported	white
TCGA-BR-4280	TCGA-STAD	Stomach	Female	78	10	4	16	11	6	8	18	832	796	4	TCGA	Adenomas and Adenocarcinomas	78 years 26 days	201 days	Dead	Adenocarcinoma, NOS	not hispanic or latino	white
TCGA-VQ-AA6D	TCGA-STAD	Stomach	Female	73	10	4	16	11	3	8	16	827	794	2	TCGA	Adenomas and Adenocarcinomas	52 years 97 days	--	Alive	Tubular adenocarcinoma	not reported	not reported

TCGA-CD-A4MJ	TCGA-STAD	Stomach	Male	71	8	4	16	11	3	8	16	799	767	2	TCGA	Adenomas and Adenocarcinomas	60 years 17 days	--	Alive	Adenocarcinoma, NOS	not hispanic or latino	asian
TCGA-BR-8059	TCGA-STAD	Stomach	Male	72	8	4	16	11	3	8	17	806	761	3	TCGA	Adenomas and Adenocarcinomas	72 years 176 days	1 year 74 days	Dead	Adenocarcinoma, intestinal type	not hispanic or latino	white
TCGA-HU-A4GH	TCGA-STAD	Stomach	Male	93	16	10	17	14	3	8	17	797	741	3	TCGA	Adenomas and Adenocarcinomas	75 years 80 days	--	Alive	Tubular adenocarcinoma	not hispanic or latino	asian

TCGA-BR-7703	TCGA-STAD	Stomach	Male	90	13	8	17	14	3	8	18	748	712	4	TCGA	Adenomas and Adenocarcinomas	81 years 126 days	--	Alive	Papillary adenocarcinoma, NOS	not hispanic or latino	white
TCGA-CG-4465	TCGA-STAD	Stomach	Female	74	10	4	16	11	3	8	17	736	700	3	TCGA	Adenomas and Adenocarcinomas	69 years 31 days	274 days	Dead	Carcinoma, diffuse type	not reported	not reported
TCGA-SW-A7EA	TCGA-STAD	Stomach	Female	75	9	6	16	11	3	8	17	724	695	3	TCGA	Cystic, Mucinous and Serous Neoplasms	61 years 14 days	--	Alive	Signet ring cell carcinoma	not hispanic or latino	white

TCGA-HU-A4H4	TCGA-STAD	Stomach	Female	71	8	4	16	11	3	8	16	724	691	2	TCGA	Adenomas and Adenocarcinomas	53 years 307 days	--	Alive	Tubular adenocarcinoma	not hispanic or latino	asian
TCGA-B7-A5TI	TCGA-STAD	Stomach	Male	72	10	4	16	11	3	8	15	710	682	1	TCGA	Adenomas and Adenocarcinomas	52 years 21 days	--	Alive	Carcinoma, diffuse type	not hispanic or latino	white
TCGA-VQ-A8PX	TCGA-STAD	Stomach	Male	73	10	4	16	11	3	8	16	702	673	2	TCGA	Adenomas and Adenocarcinomas	51 years 275 days	--	Alive	Adenocarcinoma, intestinal type	not reported	not reported

TCGA-BR-4363	TCGA-STAD	Stomach	Female	79	10	4	16	11	6	8	19	704	673	5	TCGA	Adenomas and Adenocarcinomas	60 years 281 days	--	Alive	Adenocarcinoma, NOS	not hispanic or latino	white
TCGA-RD-A8NB	TCGA-STAD	Stomach	Female	68	8	4	16	8	3	8	16	686	667	2	TCGA	Adenomas and Adenocarcinomas	80 years 283 days	1 year 148 days	Dead	Carcinoma, diffuse type	not hispanic or latino	white
TCGA-CG-4437	TCGA-STAD	Stomach	Male	72	8	4	16	11	3	8	17	576	547	3	TCGA	Adenomas and Adenocarcinomas	83 years 152 days	--	Alive	Adenocarcinoma, intestinal type	not reported	not reported

TCGA-F1-A448	TCGA-STAD	Stomach	Male	69	8	4	16	9	3	8	16	564	544	2	TCGA	Cystic, Mucinous and Serous Neoplasms	70 years 279 days	--	Alive	Mucinous adenocarcinoma	hispanic or latino	white
TCGA-D7-A4YY	TCGA-STAD	Stomach	Male	71	8	4	16	11	3	8	16	559	541	2	TCGA	Adenomas and Adenocarcinomas	61 years 7 days	--	Alive	Adenocarcinoma, NOS	not hispanic or latino	white
TCGA-BR-6802	TCGA-STAD	Stomach	Male	94	17	8	17	14	3	8	18	516	499	4	TCGA	Adenomas and Adenocarcinomas	65 years 174 days	--	Alive	Adenocarcinoma, NOS	not hispanic or latino	white

TCGA-IN-A6RL	TCGA-STAD	Stomach	Male	72	10	4	16	11	3	8	15	482	466	1	TCGA	Adenomas and Adenocarcinomas	84 years 102 days	1 year 41 days	Dead	Adenocarcinoma, NOS	not hispanic or latino	white
TCGA-CD-5801	TCGA-STAD	Stomach	Male	74	10	4	16	11	3	8	17	428	411	3	TCGA	Adenomas and Adenocarcinomas	--	1 year 36 days	Dead	Adenocarcinoma, NOS	not hispanic or latino	asian
TCGA-CD-8529	TCGA-STAD	Stomach	Male	74	10	4	16	11	3	8	17	427	409	3	TCGA	Cystic, Mucinous and Serous Neoplasms	65 years 238 days	--	Alive	Mucinous adenocarcinoma	not hispanic or latino	asian

TCGA-B7-5818	TCGA-STAD	Stomach	Male	73	10	4	16	11	3	8	16	413	397	2	TCGA	Adenomas and Adenocarcinomas	62 years 147 days	--	Alive	Carcinoma, diffuse type	not hispanic or latino	white
TCGA-BR-7704	TCGA-STAD	Stomach	Female	90	13	8	17	14	3	8	18	395	371	4	TCGA	Adenomas and Adenocarcinomas	69 years 98 days	--	Alive	Adenocarcinoma, intestinal type	not hispanic or latino	white
TCGA-D7-6528	TCGA-STAD	Stomach	Female	77	12	4	16	11	3	8	18	346	334	4	TCGA	Adenomas and Adenocarcinomas	70 years 248 days	--	Alive	Tubular adenocarcinoma	not hispanic or latino	white

TCGA-BR-8284	TCGA-STAD	Stomach	Female	72	8	4	16	11	3	8	17	327	315	3	TCGA	Adenomas and Adenocarcinomas	72 years 228 days	245 days	Dead	Adenocarcinoma, intestinal type	not hispanic or latino	white
TCGA-BR-8485	TCGA-STAD	Stomach	Female	70	8	4	16	9	3	8	17	335	315	3	TCGA	Adenomas and Adenocarcinomas	68 years 8 days	--	Alive	Adenocarcinoma, NOS	not hispanic or latino	white
TCGA-D7-A4YT	TCGA-STAD	Stomach	Male	71	8	4	16	11	3	8	16	323	314	2	TCGA	Adenomas and Adenocarcinomas	56 years 288 days	--	Alive	Tubular adenocarcinoma	not hispanic or latino	white

TCGA-BR-6458	TCGA-STAD	Stomach	Female	92	15	8	17	14	3	8	18	324	310	4	TCGA	Adenomas and Adenocarcinomas	57 years 250 days	1 year 223 days	Dead	Adenocarcinoma, NOS	not hispanic or latino	white
TCGA-VQ-A94R	TCGA-STAD	Stomach	Male	71	8	4	16	11	3	8	16	325	310	2	TCGA	Adenomas and Adenocarcinomas	63 years 236 days	3 years 199 days	Dead	Adenocarcinoma, intestinal type	not reported	white
TCGA-IN-A6RO	TCGA-STAD	Stomach	Male	72	10	4	16	11	3	8	15	305	301	1	TCGA	Adenomas and Adenocarcinomas	70 years 222 days	--	Dead	Adenocarcinoma, NOS	not hispanic or latino	asian

TCGA-CG-4469	TCGA-STAD	Stomach	Male	74	10	4	16	11	3	8	17	328	300	3	TCGA	Adenomas and Adenocarcinomas	70 years 31 days	215 days	Dead	Adenocarcinoma, intestinal type	not reported	not reported
TCGA-HU-A4H5	TCGA-STAD	Stomach	Male	70	6	4	16	11	3	8	17	297	295	3	TCGA	Adenomas and Adenocarcinomas	71 years 300 days	--	Alive	Papillary adenocarcinoma, NOS	not hispanic or latino	asian
TCGA-VQ-AA64	TCGA-STAD	Stomach	Male	73	10	4	16	11	3	8	16	300	291	2	TCGA	Adenomas and Adenocarcinomas	68 years 272 days	1 year 195 days	Dead	Adenocarcinoma, intestinal type	not reported	white

TCGA-D7-A6EZ	TCGA-STAD	Stomach	Male	80	11	4	18	13	3	8	16	293	285	2	TCGA	Adenomas and Adenocarcinomas	66 years 319 days	1 year 253 days	Dead	Adenocarcinoma, intestinal type	not hispanic or latino	white
TCGA-VQ-A8P3	TCGA-STAD	Stomach	Male	73	10	4	16	11	3	8	16	302	282	2	TCGA	Adenomas and Adenocarcinomas	72 years 284 days	--	Alive	Tubular adenocarcinoma	not reported	white
TCGA-D7-A4Z0	TCGA-STAD	Stomach	Female	69	8	4	16	9	3	8	16	325	281	2	TCGA	Cystic, Mucinous and Serous Neoplasms	60 years 214 days	--	Alive	Mucinous adenocarcinoma	not hispanic or latino	white

TCGA-HF-7134	TCGA-STAD	Stomach	Male	74	10	4	16	11	3	8	17	298	276	3	TCGA	Adenomas and Adenocarcinomas	--	--	Alive	Adenocarcinoma, intestinal type	not reported	not reported
TCGA-VQ-A91S	TCGA-STAD	Stomach	Male	73	10	4	16	11	3	8	16	275	272	2	TCGA	Adenomas and Adenocarcinomas	63 years 177 days	--	Alive	Tubular adenocarcinoma	not reported	black or african american
TCGA-CD-8527	TCGA-STAD	Stomach	Female	72	8	4	16	11	3	8	17	278	269	3	TCGA	Adenomas and Adenocarcinomas	72 years 205 days	218 days	Dead	Adenocarcinoma, intestinal type	not hispanic or latino	asian

TCGA-RD-A7C1	TCGA-STAD	Stomach	Male	73	10	4	16	11	3	8	16	281	269	2	TCGA	Adenomas and Adenocarcinomas	82 years 221 days	1 year 142 days	Dead	Carcinoma, diffuse type	not hispanic or latino	white
TCGA-FP-7829	TCGA-STAD	Stomach	Male	90	15	8	17	14	3	8	17	269	265	3	TCGA	Cystic, Mucinous and Serous Neoplasms	69 years 332 days	--	Alive	Mucinous adenocarcinoma	not hispanic or latino	white
TCGA-CD-8531	TCGA-STAD	Stomach	Female	74	10	4	16	11	3	8	17	261	244	3	TCGA	Adenomas and Adenocarcinomas	66 years 347 days	--	Alive	Carcinoma, diffuse type	not hispanic or latino	asian

TCGA-VQ-A91V	TCGA-STAD	Stomach	Male	73	10	4	16	11	3	8	16	256	238	2	TCGA	Adenomas and Adenocarcinomas	58 years 321 days	--	Alive	Tubular adenocarcinoma	not reported	not reported
TCGA-B7-A5TJ	TCGA-STAD	Stomach	Male	72	10	4	16	11	3	8	15	252	238	1	TCGA	Adenomas and Adenocarcinomas	74 years 29 days	--	Alive	Adenocarcinoma, intestinal type	not hispanic or latino	white
TCGA-RD-A7BT	TCGA-STAD	Stomach	Male	68	7	2	16	11	3	8	16	245	236	2	TCGA	Adenomas and Adenocarcinomas	66 years 1 day	262 days	Dead	Adenocarcinoma, intestinal type	not hispanic or latino	native hawaiian or other pacific islander

TCGA-BR-4357	TCGA-STAD	Stomach	Male	79	10	4	16	11	6	8	19	247	234	5	TCGA	Adenomas and Adenocarcinomas	58 years 111 days	--	Alive	Adenocarcinoma, NOS	not hispanic or latino	white
TCGA-CD-A48A	TCGA-STAD	Stomach	Male	76	11	6	16	11	3	8	16	244	234	2	TCGA	Adenomas and Adenocarcinomas	57 years 20 days	--	Alive	Adenocarcinoma, NOS	not hispanic or latino	asian
TCGA-D7-6822	TCGA-STAD	Stomach	Male	76	11	4	16	11	3	8	18	260	234	4	TCGA	Adenomas and Adenocarcinomas	77 years 90 days	--	Alive	Tubular adenocarcinoma	not hispanic or latino	white

TCGA-BR-8060	TCGA-STAD	Stomach	Female	89	12	8	17	14	3	8	18	238	226	4	TCGA	Adenomas and Adenocarcinomas	75 years 168 days	348 days	Dead	Adenocarcinoma, NOS	not hispanic or latino	white
TCGA-D7-5578	TCGA-STAD	Stomach	Male	75	10	4	16	11	3	8	18	239	225	4	TCGA	Adenomas and Adenocarcinomas	80 years 170 days	--	Alive	Tubular adenocarcinoma	not hispanic or latino	white
TCGA-BR-A4PE	TCGA-STAD	Stomach	Female	71	8	4	16	11	3	8	16	230	224	2	TCGA	Adenomas and Adenocarcinomas	68 years 149 days	--	Alive	Adenocarcinoma, NOS	not hispanic or latino	white

TCGA-CD-A48C	TCGA-STAD	Stomach	Female	78	9	4	18	13	3	8	16	233	224	2	TCGA	Adenomas and Adenocarcinomas	79 years 349 days	353 days	Dead	Adenocarcinoma, NOS	not hispanic or latino	asian
TCGA-KB-A93H	TCGA-STAD	Stomach	Female	70	9	2	16	11	3	8	16	232	223	2	TCGA	Adenomas and Adenocarcinomas	79 years 112 days	--	Alive	Tubular adenocarcinoma	not reported	asian
TCGA-HU-8604	TCGA-STAD	Stomach	Female	78	11	4	17	14	3	8	17	230	222	3	TCGA	Adenomas and Adenocarcinomas	82 years 228 days	--	Alive	Tubular adenocarcinoma	not hispanic or latino	asian

TCGA-BR-4191	TCGA-STAD	Stomach	Male	79	10	4	16	11	6	8	19	230	222	5	TCGA	Adenomas and Adenocarcinomas	72 years 264 days	1 year 193 days	Dead	Adenocarcinoma, NOS	not hispanic or latino	white
TCGA-BR-8297	TCGA-STAD	Stomach	Male	70	8	4	16	9	3	8	17	235	222	3	TCGA	Adenomas and Adenocarcinomas	58 years 40 days	--	Alive	Carcinoma, diffuse type	not hispanic or latino	white
TCGA-VQ-A91X	TCGA-STAD	Stomach	Male	73	10	4	16	11	3	8	16	230	221	2	TCGA	Adenomas and Adenocarcinomas	74 years 286 days	289 days	Dead	Adenocarcinoma, intestinal type	not reported	white

TCGA-D7-A4YU	TCGA-STAD	Stomach	Male	73	10	4	16	11	3	8	16	226	217	2	TCGA	Adenomas and Adenocarcinomas	73 years 118 days	--	Alive	Tubular adenocarcinoma	not hispanic or latino	white
TCGA-D7-6526	TCGA-STAD	Stomach	Female	74	10	4	16	11	3	8	18	225	216	4	TCGA	Adenomas and Adenocarcinomas	67 years 6 days	--	Alive	Tubular adenocarcinoma	not hispanic or latino	white
TCGA-D7-6527	TCGA-STAD	Stomach	Male	77	12	4	16	11	3	8	18	226	213	4	TCGA	Adenomas and Adenocarcinomas	62 years 148 days	312 days	Dead	Papillary adenocarcinoma, NOS	not hispanic or latino	white

TCGA-CG-4466	TCGA-STAD	Stomach	Female	74	10	4	16	11	3	8	17	214	212	3	TCGA	Adenomas and Adenocarcinomas	81 years 154 days	--	Alive	Adenocarcinoma, intestinal type	not reported	not reported
TCGA-VQ-A8P5	TCGA-STAD	Stomach	Male	73	10	4	16	11	3	8	16	219	211	2	TCGA	Adenomas and Adenocarcinomas	67 years 100 days	235 days	Dead	Adenocarcinoma, intestinal type	not reported	white
TCGA-BR-8369	TCGA-STAD	Stomach	Female	72	8	4	16	11	3	8	17	214	210	3	TCGA	Adenomas and Adenocarcinomas	76 years 292 days	--	Alive	Adenocarcinoma, NOS	not hispanic or latino	white

TCGA-VQ-A8PH	TCGA-STAD	Stomach	Male	73	10	4	16	11	3	8	16	214	203	2	TCGA	Adenomas and Adenocarcinomas	62 years 295 days	1 year 24 days	Dead	Tubular adenocarcinoma	not reported	white
TCGA-HU-A4HD	TCGA-STAD	Stomach	Male	71	8	4	16	11	3	8	16	203	197	2	TCGA	Adenomas and Adenocarcinomas	73 years 36 days	--	Alive	Tubular adenocarcinoma	not hispanic or latino	asian
TCGA-BR-6706	TCGA-STAD	Stomach	Male	82	13	4	17	14	3	8	18	200	196	4	TCGA	Adenomas and Adenocarcinomas	63 years 43 days	1 year 184 days	Dead	Adenocarcinoma, NOS	not hispanic or latino	white

TCGA-D7-8572	TCGA-STAD	Stomach	Male	73	8	4	16	11	3	8	18	198	193	4	TCGA	Adenomas and Adenocarcinomas	57 years 51 days	--	Alive	Adenocarcinoma, NOS	not hispanic or latino	white
TCGA-3M-AB46	TCGA-STAD	Stomach	Male	72	10	4	16	11	3	8	16	203	193	2	TCGA	Adenomas and Adenocarcinomas	--	--	Alive	Adenocarcinoma, NOS	hispanic or latino	white
TCGA-EQ-A4SO	TCGA-STAD	Stomach	Male	78	9	4	17	14	3	9	17	194	191	3	TCGA	Adenomas and Adenocarcinomas	70 years 330 days	--	Alive	Adenocarcinoma, intestinal type	not hispanic or latino	white

TCGA-HU-A4GP	TCGA-STAD	Stomach	Female	88	13	8	17	14	3	8	17	201	191	3	TCGA	Adenomas and Adenocarcinomas	62 years 66 days	--	Alive	Carcinoma, diffuse type	not hispanic or latino	asian
TCGA-BR-8687	TCGA-STAD	Stomach	Female	68	8	4	16	7	3	8	17	196	190	3	TCGA	Adenomas and Adenocarcinomas	67 years 1 day	250 days	Dead	Adenocarcinoma, NOS	not hispanic or latino	white
TCGA-BR-6453	TCGA-STAD	Stomach	Male	85	14	8	16	9	3	8	18	195	185	4	TCGA	Adenomas and Adenocarcinomas	54 years 338 days	--	Alive	Adenocarcinoma, NOS	not hispanic or latino	white

TCGA-HU-A4H0	TCGA-STAD	Stomach	Male	71	10	4	16	9	3	8	17	188	184	3	TCGA	Adenomas and Adenocarcinomas	72 years 19 days	--	Alive	Carcinoma, diffuse type	not hispanic or latino	asian
TCGA-BR-A4CQ	TCGA-STAD	Stomach	Male	63	6	4	16	9	3	8	16	191	183	2	TCGA	Adenomas and Adenocarcinomas	58 years 23 days	--	Alive	Adenocarcinoma, NOS	not hispanic or latino	white
TCGA-CD-A487	TCGA-STAD	Stomach	Male	71	8	4	16	11	3	8	16	189	181	2	TCGA	Adenomas and Adenocarcinomas	51 years 67 days	--	Alive	Carcinoma, diffuse type	not hispanic or latino	asian

TCGA-BR-8366	TCGA-STAD	Stomach	Female	70	8	4	16	9	3	8	17	185	181	3	TCGA	Adenomas and Adenocarcinomas	80 years 174 days	--	Alive	Adenocarcinoma, NOS	not hispanic or latino	white
TCGA-HU-8238	TCGA-STAD	Stomach	Male	89	14	8	17	14	3	8	17	184	180	3	TCGA	Adenomas and Adenocarcinomas	56 years 254 days	--	Alive	Tubular adenocarcinoma	not hispanic or latino	asian
TCGA-D7-6525	TCGA-STAD	Stomach	Male	73	10	4	16	9	3	8	18	181	180	4	TCGA	Adenomas and Adenocarcinomas	58 years 39 days	1 year 41 days	Dead	Carcinoma, diffuse type	not hispanic or latino	white

TCGA-BR-4369	TCGA-STAD	Stomach	Male	79	10	4	16	11	6	8	19	184	179	5	TCGA	Adenomas and Adenocarcinomas	75 years 116 days	--	Alive	Adenocarcinoma, NOS	not hispanic or latino	white
TCGA-BR-7958	TCGA-STAD	Stomach	Male	71	8	4	16	11	3	8	16	181	179	2	TCGA	Adenomas and Adenocarcinomas	60 years 289 days	--	Alive	Adenocarcinoma, NOS	not hispanic or latino	white
TCGA-VQ-A8DT	TCGA-STAD	Stomach	Male	71	8	4	16	11	3	8	16	182	179	2	TCGA	Adenomas and Adenocarcinomas	43 years 232 days	--	Alive	Adenocarcinoma, intestinal type	not reported	not reported

TCGA-BR-7197	TCGA-STAD	Stomach	Male	78	9	4	17	14	3	8	18	186	177	4	TCGA	Adenomas and Adenocarcinomas	69 years 243 days	--	Alive	Adenocarcinoma, NOS	not hispanic or latino	white
TCGA-EQ-8122	TCGA-STAD	Stomach	Female	74	8	4	17	12	3	8	17	188	175	3	TCGA	Adenomas and Adenocarcinomas	71 years 357 days	243 days	Dead	Adenocarcinoma, intestinal type	not hispanic or latino	white
TCGA-BR-7723	TCGA-STAD	Stomach	Male	74	10	4	16	11	3	8	17	179	175	3	TCGA	Adenomas and Adenocarcinomas	59 years 290 days	2 years 144 days	Dead	Adenocarcinoma, NOS	not hispanic or latino	white

TCGA-HU-A4H2	TCGA-STAD	Stomach	Female	70	8	4	16	11	3	8	15	182	175	1	TCGA	Adenomas and Adenocarcinomas	58 years 312 days	--	Alive	Tubular adenocarcinoma	not hispanic or latino	asian
TCGA-CD-5813	TCGA-STAD	Stomach	Male	69	10	4	16	6	3	8	17	177	174	3	TCGA	Cystic, Mucinous and Serous Neoplasms	60 years 80 days	1 year 12 days	Dead	Mucinous adenocarcinoma	not hispanic or latino	asian
TCGA-VQ-A8DL	TCGA-STAD	Stomach	Female	62	5	2	16	11	3	8	16	172	171	2	TCGA	Adenomas and Adenocarcinomas	69 years 97 days	28 days	Dead	Tubular adenocarcinoma	not reported	white

TCGA-VQ-AA68	TCGA-STAD	Stomach	Female	72	10	4	16	11	3	8	16	181	171	2	TCGA	Adenomas and Adenocarcinomas	52 years 311 days	--	Alive	Tubular adenocarcinoma	not reported	white
TCGA-CG-5730	TCGA-STAD	Stomach	Female	80	13	6	16	11	3	8	18	171	170	4	TCGA	Adenomas and Adenocarcinomas	80 years 212 days	--	Alive	Adenocarcinoma, intestinal type	not reported	not reported
TCGA-IN-8663	TCGA-STAD	Stomach	Male	92	15	8	17	14	3	8	18	172	169	4	TCGA	Adenomas and Adenocarcinomas	68 years 335 days	103 days	Dead	Adenocarcinoma, NOS	not hispanic or latino	white

TCGA-VQ-A8PJ	TCGA-STAD	Stomach	Male	78	9	4	18	13	3	8	16	172	168	2	TCGA	Adenomas and Adenocarcinomas	53 years 32 days	82 days	Dead	Tubular adenocarcinoma	not reported	not reported
TCGA-CG-4444	TCGA-STAD	Stomach	Male	75	10	4	16	11	3	9	17	178	168	3	TCGA	Adenomas and Adenocarcinomas	76 years 153 days	--	Alive	Carcinoma, diffuse type	not reported	not reported
TCGA-RD-A8N6	TCGA-STAD	Stomach	Female	72	10	4	16	11	3	8	16	171	166	2	TCGA	Adenomas and Adenocarcinomas	78 years 345 days	272 days	Dead	Adenocarcinoma, intestinal type	not hispanic or latino	white

TCGA-CG-5727	TCGA-STAD	Stomach	Male	67	7	2	16	11	3	9	18	172	165	4	TCGA	Adenomas and Adenocarcinomas	66 years 183 days	--	Alive	Adenocarcinoma, intestinal type	not reported	not reported
TCGA-BR-A4PD	TCGA-STAD	Stomach	Female	73	10	4	16	11	3	8	16	166	164	2	TCGA	Adenomas and Adenocarcinomas	72 years 36 days	--	Alive	Adenocarcinoma, NOS	not hispanic or latino	white
TCGA-D7-6818	TCGA-STAD	Stomach	Male	77	12	4	16	11	3	8	18	169	164	4	TCGA	Adenomas and Adenocarcinomas	53 years 33 days	1 year 11 days	Dead	Carcinoma, diffuse type	not hispanic or latino	white

TCGA-HU-8249	TCGA-STAD	Stomach	Male	73	9	6	16	11	3	8	16	165	163	2	TCGA	Adenomas and Adenocarcinomas	76 years 253 days	--	Alive	Tubular adenocarcinoma	not hispanic or latino	asian
TCGA-BR-7901	TCGA-STAD	Stomach	Male	70	10	4	16	7	3	8	17	166	163	3	TCGA	Adenomas and Adenocarcinomas	74 years 3 days	105 days	Dead	Adenocarcinoma, NOS	not hispanic or latino	white
TCGA-KB-A93J	TCGA-STAD	Stomach	Male	70	8	4	16	11	3	8	16	163	161	2	TCGA	Adenomas and Adenocarcinomas	78 years 110 days	--	Alive	Carcinoma, diffuse type	not reported	white

TCGA-CD-5800	TCGA-STAD	Stomach	Female	74	10	4	16	11	3	8	17	163	160	3	TCGA	Adenomas and Adenocarcinomas	--	--	Alive	Papillary adenocarcinoma, NOS	not hispanic or latino	asian
TCGA-D7-A6EV	TCGA-STAD	Stomach	Female	73	10	4	16	11	3	8	16	165	160	2	TCGA	Adenomas and Adenocarcinomas	71 years 294 days	--	Alive	Adenocarcinoma, NOS	not hispanic or latino	white
TCGA-BR-8690	TCGA-STAD	Stomach	Female	71	9	4	16	9	3	8	17	167	159	3	TCGA	Adenomas and Adenocarcinomas	54 years 132 days	--	Alive	Adenocarcinoma, NOS	not hispanic or latino	white

TCGA-VQ-A94U	TCGA-STAD	Stomach	Male	72	8	4	16	11	3	9	16	163	159	2	TCGA	Cystic, Mucinous and Serous Neoplasms	70 years 358 days	--	Alive	Signet ring cell carcinoma	not reported	white
TCGA-FP-A9TM	TCGA-STAD	Stomach	Male	72	10	4	16	11	3	8	16	162	159	2	TCGA	Adenomas and Adenocarcinomas	77 years 252 days	--	Alive	Adenocarcinoma, NOS	not hispanic or latino	white
TCGA-CG-4436	TCGA-STAD	Stomach	Male	74	10	4	16	11	3	8	17	167	158	3	TCGA	Adenomas and Adenocarcinomas	57 years 122 days	--	Alive	Adenocarcinoma, intestinal type	not reported	not reported

TCGA-R5-A7ZE	TCGA-STAD	Stomach	Female	69	8	4	16	11	3	8	15	160	157	1	TCGA	Adenomas and Adenocarcinomas	66 years 222 days	1 year 189 days	Dead	Tubular adenocarcinoma	not hispanic or latino	white
TCGA-IN-A6RP	TCGA-STAD	Stomach	Male	62	7	2	16	11	3	8	15	165	157	1	TCGA	Adenomas and Adenocarcinomas	63 years 272 days	--	Alive	Adenocarcinoma, NOS	not hispanic or latino	white
TCGA-VQ-A91Z	TCGA-STAD	Stomach	Female	73	10	4	16	11	3	8	16	161	157	2	TCGA	Adenomas and Adenocarcinomas	67 years 181 days	--	Alive	Tubular adenocarcinoma	not reported	white

TCGA-BR-8080	TCGA-STAD	Stomach	Female	72	8	4	16	11	3	8	17	164	157	3	TCGA	Cystic, Mucinous and Serous Neoplasms	72 years 248 days	292 days	Dead	Mucinous adenocarcinoma	not hispanic or latino	white
TCGA-VQ-A91U	TCGA-STAD	Stomach	Male	71	8	4	16	11	3	8	16	158	156	2	TCGA	Adenomas and Adenocarcinomas	78 years 302 days	52 days	Dead	Adenocarcinoma, intestinal type	not reported	asian
TCGA-BR-4371	TCGA-STAD	Stomach	Female	77	8	4	16	11	6	8	19	160	156	5	TCGA	Adenomas and Adenocarcinomas	71 years 358 days	--	Alive	Adenocarcinoma, NOS	not hispanic or latino	white

TCGA-BR-A4CS	TCGA-STAD	Stomach	Male	78	9	4	18	13	3	8	16	157	155	2	TCGA	Adenomas and Adenocarcinomas	77 years 87 days	45 days	Dead	Adenocarcinoma, NOS	not hispanic or latino	white
TCGA-BR-8286	TCGA-STAD	Stomach	Male	72	8	4	16	11	3	8	17	157	154	3	TCGA	Adenomas and Adenocarcinomas	49 years 69 days	--	Alive	Adenocarcinoma, NOS	not hispanic or latino	white
TCGA-BR-7716	TCGA-STAD	Stomach	Female	91	15	8	17	14	3	8	17	161	153	3	TCGA	Adenomas and Adenocarcinomas	62 years 184 days	--	Alive	Adenocarcinoma, NOS	not hispanic or latino	white

TCGA-IN-A6RR	TCGA-STAD	Stomach	Male	72	10	4	16	11	3	8	15	156	152	1	TCGA	Adenomas and Adenocarcinomas	84 years 53 days	205 days	Dead	Adenocarcinoma, NOS	not hispanic or latino	white
TCGA-D7-A74A	TCGA-STAD	Stomach	Female	72	10	4	16	11	3	8	16	156	151	2	TCGA	Adenomas and Adenocarcinomas	61 years 155 days	--	Alive	Adenocarcinoma, intestinal type	not hispanic or latino	white
TCGA-BR-8683	TCGA-STAD	Stomach	Male	72	8	4	16	11	3	8	17	155	150	3	TCGA	Adenomas and Adenocarcinomas	75 years 156 days	300 days	Dead	Tubular adenocarcinoma	not hispanic or latino	asian

TCGA-BR-6454	TCGA-STAD	Stomach	Male	87	14	8	16	11	3	8	18	154	150	4	TCGA	Adenomas and Adenocarcinomas	58 years 317 days	--	Alive	Adenocarcinoma, NOS	not hispanic or latino	white
TCGA-VQ-A922	TCGA-STAD	Stomach	Male	72	10	4	16	11	3	8	16	151	150	2	TCGA	Adenomas and Adenocarcinomas	70 years 133 days	275 days	Dead	Tubular adenocarcinoma	not reported	not reported
TCGA-HU-A4GC	TCGA-STAD	Stomach	Male	88	13	8	17	14	3	8	17	152	149	3	TCGA	Adenomas and Adenocarcinomas	74 years 239 days	--	Alive	Carcinoma, diffuse type	not hispanic or latino	asian

TCGA-D7-5579	TCGA-STAD	Stomach	Male	68	9	2	16	11	3	8	18	152	149	4	TCGA	Cystic, Mucinous and Serous Neoplasms	74 years 147 days	--	Alive	Mucinous adenocarcinoma	not hispanic or latino	white
TCGA-CD-8524	TCGA-STAD	Stomach	Female	74	10	4	16	11	3	8	17	155	148	3	TCGA	Adenomas and Adenocarcinomas	61 years 165 days	--	Alive	Adenocarcinoma, intestinal type	not hispanic or latino	asian
TCGA-BR-A4IY	TCGA-STAD	Stomach	Male	72	9	4	16	11	3	8	16	150	147	2	TCGA	Adenomas and Adenocarcinomas	58 years 130 days	--	Alive	Adenocarcinoma, NOS	not hispanic or latino	asian

TCGA-HU-A4GF	TCGA-STAD	Stomach	Male	71	8	4	16	11	3	8	16	149	146	2	TCGA	Adenomas and Adenocarcinomas	69 years 21 days	--	Alive	Tubular adenocarcinoma	not hispanic or latino	asian
TCGA-FP-8099	TCGA-STAD	Stomach	Male	74	8	4	17	14	3	8	16	152	146	2	TCGA	Adenomas and Adenocarcinomas	79 years 275 days	--	Alive	Adenocarcinoma, NOS	not hispanic or latino	white
TCGA-CG-4438	TCGA-STAD	Stomach	Male	72	8	4	16	11	3	8	17	146	145	3	TCGA	Adenomas and Adenocarcinomas	56 years 120 days	--	Alive	Adenocarcinoma, intestinal type	not reported	not reported

TCGA-BR-4366	TCGA-STAD	Stomach	Male	79	10	4	16	11	6	8	19	150	145	5	TCGA	Adenomas and Adenocarcinomas	87 years 248 days	--	Alive	Adenocarcinoma, NOS	not hispanic or latino	white
TCGA-VQ-AA6J	TCGA-STAD	Stomach	Male	70	10	4	16	9	3	8	16	147	144	2	TCGA	Adenomas and Adenocarcinomas	75 years 113 days	--	Alive	Adenocarcinoma, intestinal type	not reported	black or african american
TCGA-VQ-A8DU	TCGA-STAD	Stomach	Male	73	10	4	16	11	3	8	16	145	144	2	TCGA	Adenomas and Adenocarcinomas	63 years 32 days	166 days	Dead	Tubular adenocarcinoma	not reported	white

TCGA-BR-7715	TCGA-STAD	Stomach	Male	92	15	8	17	14	3	8	18	143	143	4	TCGA	Adenomas and Adenocarcinomas	65 years 120 days	--	Alive	Adenocarcinoma, NOS	not hispanic or latino	white
TCGA-VQ-A92D	TCGA-STAD	Stomach	Male	73	10	4	16	11	3	8	16	143	142	2	TCGA	Adenomas and Adenocarcinomas	67 years 124 days	--	Alive	Tubular adenocarcinoma	not reported	white
TCGA-CG-4477	TCGA-STAD	Stomach	Female	74	10	4	16	11	3	8	17	141	139	3	TCGA	Adenomas and Adenocarcinomas	58 years 243 days	--	Alive	Adenocarcinoma, intestinal type	not reported	not reported

TCGA-BR-7717	TCGA-STAD	Stomach	Male	90	13	8	17	14	3	8	18	143	139	4	TCGA	Adenomas and Adenocarcinomas	63 years 92 days	1 year 187 days	Dead	Adenocarcinoma, NOS	not hispanic or latino	white
TCGA-D7-6815	TCGA-STAD	Stomach	Female	76	12	4	16	11	3	8	17	142	139	3	TCGA	Adenomas and Adenocarcinomas	70 years 244 days	--	Alive	Tubular adenocarcinoma	not hispanic or latino	white
TCGA-BR-6565	TCGA-STAD	Stomach	Male	74	10	4	16	11	3	8	17	140	136	3	TCGA	Adenomas and Adenocarcinomas	67 years 24 days	279 days	Dead	Adenocarcinoma, NOS	not hispanic or latino	white

TCGA-D7-A6F0	TCGA-STAD	Stomach	Female	73	10	4	16	11	3	8	16	136	136	2	TCGA	Adenomas and Adenocarcinomas	79 years 69 days	--	Alive	Adenocarcinoma, intestinal type	not hispanic or latino	white
TCGA-SW-A7EB	TCGA-STAD	Stomach	Male	73	10	4	16	11	3	8	16	138	135	2	TCGA	Cystic, Mucinous and Serous Neoplasms	45 years 39 days	--	Alive	Mucinous adenocarcinoma	not hispanic or latino	white
TCGA-CG-4440	TCGA-STAD	Stomach	Female	73	10	4	16	11	3	8	17	140	135	3	TCGA	Adenomas and Adenocarcinomas	68 years 334 days	122 days	Dead	Adenocarcinoma, intestinal type	not reported	not reported

TCGA-VQ-A8PU	TCGA-STAD	Stomach	Female	74	10	4	16	11	3	9	16	140	135	2	TCGA	Adenomas and Adenocarcinomas	72 years 327 days	2 years 102 days	Dead	Tubular adenocarcinoma	not reported	white
TCGA-BR-A4QI	TCGA-STAD	Stomach	Female	71	8	4	16	11	3	8	16	135	135	2	TCGA	Adenomas and Adenocarcinomas	70 years 361 days	1 year 287 days	Dead	Adenocarcinoma, NOS	not hispanic or latino	white
TCGA-VQ-AA6G	TCGA-STAD	Stomach	Male	73	10	4	16	11	3	8	16	137	134	2	TCGA	Adenomas and Adenocarcinomas	68 years 254 days	2 years 62 days	Dead	Adenocarcinoma, intestinal type	not reported	white

TCGA-HU-8244	TCGA-STAD	Stomach	Female	73	6	4	17	14	3	8	17	136	132	3	TCGA	Adenomas and Adenocarcinomas	77 years 60 days	--	Alive	Tubular adenocarcinoma	not hispanic or latino	asian
TCGA-VQ-A8PC	TCGA-STAD	Stomach	Male	73	10	4	16	11	3	8	16	134	132	2	TCGA	Cystic, Mucinous and Serous Neoplasms	65 years 171 days	3 years 312 days	Dead	Signet ring cell carcinoma	not reported	white
TCGA-RD-A8N9	TCGA-STAD	Stomach	Female	72	10	4	16	11	3	8	16	134	132	2	TCGA	Adenomas and Adenocarcinomas	63 years 49 days	--	Alive	Carcinoma, diffuse type	not hispanic or latino	asian

TCGA-R5-A805	TCGA-STAD	Stomach	Male	69	8	4	16	11	3	8	15	134	131	1	TCGA	Adenomas and Adenocarcinomas	71 years 190 days	281 days	Dead	Adenocarcinoma, intestinal type	not hispanic or latino	white
TCGA-VQ-A91A	TCGA-STAD	Stomach	Male	72	10	4	16	11	3	8	16	134	131	2	TCGA	Cystic, Mucinous and Serous Neoplasms	67 years 199 days	--	Alive	Mucinous adenocarcinoma	not reported	white
TCGA-HU-8243	TCGA-STAD	Stomach	Male	72	8	4	17	12	3	8	16	131	130	2	TCGA	Adenomas and Adenocarcinomas	66 years 81 days	180 days	Dead	Tubular adenocarcinoma	not hispanic or latino	asian

TCGA-BR-8483	TCGA-STAD	Stomach	Male	70	8	4	16	11	3	8	15	134	128	1	TCGA	Adenomas and Adenocarcinomas	59 years 182 days	--	Alive	Adenocarcinoma, intestinal type	not hispanic or latino	white
TCGA-IN-8462	TCGA-STAD	Stomach	Male	86	12	8	17	12	3	8	17	129	128	3	TCGA	Adenomas and Adenocarcinomas	80 years 22 days	--	Alive	Adenocarcinoma, NOS	not hispanic or latino	white
TCGA-CD-8533	TCGA-STAD	Stomach	Male	72	10	4	16	9	3	8	17	126	126	3	TCGA	Adenomas and Adenocarcinomas	48 years 289 days	--	Alive	Adenocarcinoma, NOS	not hispanic or latino	asian

TCGA-VQ-A925	TCGA-STAD	Stomach	Male	73	10	4	16	11	3	8	16	130	126	2	TCGA	Adenomas and Adenocarcinomas	66 years 124 days	138 days	Dead	Tubular adenocarcinoma	not reported	white
TCGA-BR-8588	TCGA-STAD	Stomach	Female	74	10	4	16	11	3	8	17	128	125	3	TCGA	Adenomas and Adenocarcinomas	55 years 89 days	--	Alive	Adenocarcinoma, NOS	not hispanic or latino	white
TCGA-D7-A6EX	TCGA-STAD	Stomach	Female	73	10	4	16	11	3	8	16	129	125	2	TCGA	Adenomas and Adenocarcinomas	72 years 227 days	--	Alive	Tubular adenocarcinoma	not hispanic or latino	white

TCGA-IN-A7NT	TCGA-STAD	Stomach	Female	70	8	4	16	11	3	8	15	126	125	1	TCGA	Adenomas and Adenocarcinomas	73 years 64 days	--	Alive	Adenocarcinoma, NOS	not hispanic or latino	white
TCGA-BR-8370	TCGA-STAD	Stomach	Male	63	5	2	16	11	3	8	17	124	123	3	TCGA	Adenomas and Adenocarcinomas	64 years 260 days	--	Alive	Adenocarcinoma, NOS	not hispanic or latino	white
TCGA-BR-8381	TCGA-STAD	Stomach	Male	74	10	4	16	11	3	8	17	125	123	3	TCGA	Adenomas and Adenocarcinomas	51 years 216 days	--	Alive	Adenocarcinoma, NOS	not hispanic or latino	white

TCGA-3M-AB47	TCGA-STAD	Stomach	Male	71	8	4	16	11	3	8	16	126	123	2	TCGA	Adenomas and Adenocarcinomas	--	--	Dead	Adenocarcinoma, NOS	not hispanic or latino	white
TCGA-VQ-A8PE	TCGA-STAD	Stomach	Male	73	10	4	16	11	3	8	16	128	123	2	TCGA	Adenomas and Adenocarcinomas	78 years 266 days	1 year 310 days	Dead	Tubular adenocarcinoma	not reported	white
TCGA-R5-A7O7	TCGA-STAD	Stomach	Male	69	8	4	16	11	3	8	15	124	122	1	TCGA	Adenomas and Adenocarcinomas	51 years 357 days	--	Alive	Tubular adenocarcinoma	hispanic or latino	white

TCGA-IN-AB1X	TCGA-STAD	Stomach	Female	90	15	8	17	14	3	8	16	123	122	2	TCGA	Adenomas and Adenocarcinomas	78 years 4 days	--	Alive	Adenocarcinoma, NOS	not hispanic or latino	white
TCGA-D7-8570	TCGA-STAD	Stomach	Male	75	10	4	16	11	3	8	18	121	121	4	TCGA	Adenomas and Adenocarcinomas	44 years 79 days	--	Alive	Tubular adenocarcinoma	not hispanic or latino	white
TCGA-VQ-A8E2	TCGA-STAD	Stomach	Male	73	10	4	16	11	3	8	16	121	121	2	TCGA	Adenomas and Adenocarcinomas	57 years 293 days	--	Alive	Tubular adenocarcinoma	not reported	asian

TCGA-VQ-A91N	TCGA-STAD	Stomach	Female	73	10	4	16	11	3	8	16	121	120	2	TCGA	Adenomas and Adenocarcinomas	59 years 204 days	1 year 205 days	Dead	Tubular adenocarcinoma	not reported	not reported
TCGA-D7-5577	TCGA-STAD	Stomach	Female	75	10	4	16	11	3	8	18	123	119	4	TCGA	Adenomas and Adenocarcinomas	53 years 310 days	2 years 52 days	Dead	Tubular adenocarcinoma	not hispanic or latino	white
TCGA-IN-7808	TCGA-STAD	Stomach	Male	80	11	4	17	14	3	8	18	118	118	4	TCGA	Adenomas and Adenocarcinomas	59 years 20 days	105 days	Dead	Carcinoma, diffuse type	not hispanic or latino	white

TCGA-CG-4441	TCGA-STAD	Stomach	Male	73	10	4	16	11	3	8	17	118	118	3	TCGA	Adenomas and Adenocarcinomas	83 years 213 days	1 year 61 days	Dead	Adenocarcinoma, intestinal type	not reported	not reported
TCGA-CG-4476	TCGA-STAD	Stomach	Male	75	10	4	16	11	3	9	17	119	118	3	TCGA	Adenomas and Adenocarcinomas	69 years 304 days	--	Alive	Carcinoma, diffuse type	not reported	not reported
TCGA-IN-A6RN	TCGA-STAD	Stomach	Female	72	10	4	16	11	3	8	15	118	117	1	TCGA	Adenomas and Adenocarcinomas	72 years 111 days	--	Alive	Adenocarcinoma, NOS	not hispanic or latino	white

TCGA-ZQ-A9CR	TCGA-STAD	Stomach	Female	72	10	4	16	11	3	8	16	118	117	2	TCGA	Adenomas and Adenocarcinomas	79 years 253 days	24 days	Dead	Tubular adenocarcinoma	not hispanic or latino	white
TCGA-BR-8077	TCGA-STAD	Stomach	Female	68	8	4	16	9	3	8	16	120	116	2	TCGA	Adenomas and Adenocarcinomas	58 years 229 days	--	Alive	Adenocarcinoma, intestinal type	not hispanic or latino	white
TCGA-BR-8678	TCGA-STAD	Stomach	Male	72	8	4	16	11	3	8	17	120	114	3	TCGA	Adenomas and Adenocarcinomas	76 years 278 days	--	Alive	Adenocarcinoma, NOS	not hispanic or latino	white

TCGA-BR-A452	TCGA-STAD	Stomach	Male	72	9	4	16	11	3	8	16	118	114	2	TCGA	Adenomas and Adenocarcinomas	57 years 288 days	229 days	Dead	Tubular adenocarcinoma	not hispanic or latino	asian
TCGA-KB-A6F7	TCGA-STAD	Stomach	Female	68	8	4	16	9	3	8	16	116	113	2	TCGA	Adenomas and Adenocarcinomas	54 years 42 days	--	Alive	Adenocarcinoma, intestinal type	not reported	not reported
TCGA-IP-7968	TCGA-STAD	Stomach	Male	90	13	8	17	14	3	8	18	117	112	4	TCGA	Cystic, Mucinous and Serous Neoplasms	74 years 86 days	--	Alive	Adenocarcinoma, NOS	not hispanic or latino	white

TCGA-IN-A6RS	TCGA-STAD	Stomach	Male	72	10	4	16	11	3	8	15	113	112	1	TCGA	Adenomas and Adenocarcinomas	76 years 49 days	--	Alive	Adenocarcinoma, NOS	not hispanic or latino	white
TCGA-BR-A4QM	TCGA-STAD	Stomach	Male	71	8	4	16	11	3	8	16	113	111	2	TCGA	Adenomas and Adenocarcinomas	65 years 258 days	--	Alive	Adenocarcinoma, NOS	not hispanic or latino	white
TCGA-MX-A666	TCGA-STAD	Stomach	Male	72	10	4	16	11	3	8	16	112	111	2	TCGA	Adenomas and Adenocarcinomas	61 years 8 days	--	Alive	Adenocarcinoma, NOS	not hispanic or latino	white

TCGA-D7-8579	TCGA-STAD	Stomach	Female	71	8	4	16	11	3	8	17	115	111	3	TCGA	Adenomas and Adenocarcinomas	66 years 14 days	--	Alive	Adenocarcinoma, intestinal type	not hispanic or latino	white
TCGA-IN-A6RJ	TCGA-STAD	Stomach	Male	71	10	4	16	11	3	8	15	121	111	1	TCGA	Adenomas and Adenocarcinomas	64 years 63 days	--	Alive	Adenocarcinoma, NOS	not hispanic or latino	white
TCGA-BR-8590	TCGA-STAD	Stomach	Male	74	10	4	16	11	3	8	17	112	110	3	TCGA	Adenomas and Adenocarcinomas	62 years 292 days	284 days	Dead	Adenocarcinoma, NOS	not hispanic or latino	white

TCGA-IN-A6RI	TCGA-STAD	Stomach	Male	72	10	4	16	11	3	8	15	112	110	1	TCGA	Adenomas and Adenocarcinomas	45 years 352 days	--	Alive	Adenocarcinoma, NOS	not hispanic or latino	white
TCGA-CG-5724	TCGA-STAD	Stomach	Male	77	12	4	16	11	3	8	18	113	110	4	TCGA	Adenomas and Adenocarcinomas	59 years 213 days	1 year 1 day	Dead	Carcinoma, diffuse type	not reported	not reported
TCGA-VQ-A923	TCGA-STAD	Stomach	Male	71	10	4	16	9	3	8	16	110	110	2	TCGA	Adenomas and Adenocarcinomas	90 years	--	Dead	Tubular adenocarcinoma	not reported	white

TCGA-BR-A4PF	TCGA-STAD	Stomach	Male	71	8	4	16	11	3	8	16	111	110	2	TCGA	Adenomas and Adenocarcinomas	72 years 114 days	--	Alive	Adenocarcinoma, NOS	not hispanic or latino	white
TCGA-HU-A4G3	TCGA-STAD	Stomach	Male	76	10	6	17	11	3	8	17	113	109	3	TCGA	Adenomas and Adenocarcinomas	54 years 341 days	--	Alive	Tubular adenocarcinoma	not hispanic or latino	asian
TCGA-CG-4300	TCGA-STAD	Stomach	Male	60	7	2	16	6	3	8	17	109	109	3	TCGA	Adenomas and Adenocarcinomas	79 years 274 days	1 year 244 days	Dead	Adenocarcinoma, NOS	not reported	not reported

TCGA-FP-8211	TCGA-STAD	Stomach	Male	72	6	4	17	14	3	8	16	113	109	2	TCGA	Adenomas and Adenocarcinomas	62 years 312 days	--	Alive	Adenocarcinoma, NOS	not hispanic or latino	white
TCGA-VQ-A91Q	TCGA-STAD	Stomach	Male	72	10	4	16	11	3	8	16	110	108	2	TCGA	Adenomas and Adenocarcinomas	61 years 23 days	1 year 268 days	Dead	Tubular adenocarcinoma	not reported	not reported
TCGA-BR-8686	TCGA-STAD	Stomach	Male	72	8	4	16	11	3	8	17	110	108	3	TCGA	Adenomas and Adenocarcinomas	69 years 170 days	1 year 270 days	Dead	Tubular adenocarcinoma	not hispanic or latino	asian

TCGA-RD-A8N1	TCGA-STAD	Stomach	Male	73	10	4	16	11	3	8	16	108	108	2	TCGA	Adenomas and Adenocarcinomas	70 years 170 days	--	Alive	Carcinoma, diffuse type	not hispanic or latino	white
TCGA-VQ-A8E7	TCGA-STAD	Stomach	Male	73	10	4	16	11	3	8	16	109	107	2	TCGA	Adenomas and Adenocarcinomas	59 years 115 days	--	Alive	Tubular adenocarcinoma	not reported	white
TCGA-BR-6457	TCGA-STAD	Stomach	Male	91	15	8	17	14	3	8	17	109	106	3	TCGA	Adenomas and Adenocarcinomas	69 years 194 days	--	Alive	Adenocarcinoma, NOS	not hispanic or latino	white

TCGA-FP-8631	TCGA-STAD	Stomach	Male	68	6	4	16	11	3	8	16	109	106	2	TCGA	Adenomas and Adenocarcinomas	68 years 61 days	--	Alive	Adenocarcinoma, intestinal type	not hispanic or latino	white
TCGA-VQ-AA6I	TCGA-STAD	Stomach	Male	73	10	4	16	11	3	8	16	107	106	2	TCGA	Adenomas and Adenocarcinomas	68 years 254 days	1 year 126 days	Dead	Adenocarcinoma, NOS	not reported	white
TCGA-FP-A4BF	TCGA-STAD	Stomach	Male	71	7	6	16	11	3	8	16	106	105	2	TCGA	Cystic, Mucinous and Serous Neoplasms	68 years 193 days	168 days	Dead	Signet ring cell carcinoma	not hispanic or latino	white

TCGA-CG-5725	TCGA-STAD	Stomach	Male	76	9	6	16	11	3	8	18	107	105	4	TCGA	Adenomas and Adenocarcinomas	72 years 31 days	1 year 92 days	Dead	Adenocarcinoma, NOS	not reported	not reported
TCGA-HU-A4H6	TCGA-STAD	Stomach	Female	70	8	4	16	11	3	8	16	104	104	2	TCGA	Adenomas and Adenocarcinomas	72 years 147 days	--	Alive	Tubular adenocarcinoma	not hispanic or latino	asian
TCGA-CG-4443	TCGA-STAD	Stomach	Male	76	12	4	16	11	3	8	17	105	104	3	TCGA	Adenomas and Adenocarcinomas	68 years 153 days	--	Alive	Adenocarcinoma, intestinal type	not reported	not reported

TCGA-VQ-A8E0	TCGA-STAD	Stomach	Male	73	10	4	16	11	3	8	16	105	103	2	TCGA	Adenomas and Adenocarcinomas	68 years 11 days	1 year 197 days	Dead	Adenocarcinoma, intestinal type	not reported	black or african american
TCGA-BR-8285	TCGA-STAD	Stomach	Female	68	8	4	16	11	3	8	17	105	103	3	TCGA	Adenomas and Adenocarcinomas	57 years 30 days	17 days	Dead	Adenocarcinoma, intestinal type	not hispanic or latino	white
TCGA-VQ-AA6A	TCGA-STAD	Stomach	Male	71	8	4	16	11	3	8	16	104	102	2	TCGA	Adenomas and Adenocarcinomas	56 years 310 days	--	Alive	Adenocarcinoma, intestinal type	not reported	white

TCGA-IN-A7NU	TCGA-STAD	Stomach	Male	72	10	4	16	11	3	8	15	103	101	1	TCGA	Adenomas and Adenocarcinomas	69 years 313 days	--	Alive	Adenocarcinoma, NOS	not hispanic or latino	white
TCGA-CD-8528	TCGA-STAD	Stomach	Female	72	8	4	16	11	3	8	17	102	100	3	TCGA	Adenomas and Adenocarcinomas	43 years 281 days	--	Alive	Adenocarcinoma, NOS	not hispanic or latino	asian
TCGA-VQ-A8PF	TCGA-STAD	Stomach	Male	73	10	4	16	11	3	8	16	101	100	2	TCGA	Adenomas and Adenocarcinomas	76 years 357 days	76 days	Dead	Tubular adenocarcinoma	not reported	not reported

TCGA-CG-4449	TCGA-STAD	Stomach	Male	65	7	2	16	11	3	8	17	101	99	3	TCGA	Cystic, Mucinous and Serous Neoplasms	81 years 120 days	--	Alive	Mucinous adenocarcinoma	not reported	not reported
TCGA-HU-8608	TCGA-STAD	Stomach	Male	79	10	4	17	14	3	8	18	99	98	4	TCGA	Adenomas and Adenocarcinomas	70 years 222 days	--	Alive	Carcinoma, diffuse type	not hispanic or latino	asian
TCGA-VQ-A8PK	TCGA-STAD	Stomach	Male	71	8	4	16	11	3	8	16	100	98	2	TCGA	Adenomas and Adenocarcinomas	58 years 235 days	1 year 178 days	Dead	Adenocarcinoma, intestinal type	not reported	white

TCGA-HF-7131	TCGA-STAD	Stomach	Female	73	8	4	16	11	3	9	17	99	98	3	TCGA	Adenomas and Adenocarcinomas	--	--	Alive	Adenocarcinoma, NOS	not reported	not reported
TCGA-BR-7959	TCGA-STAD	Stomach	Male	77	8	4	17	14	3	8	18	97	97	4	TCGA	Cystic, Mucinous and Serous Neoplasms	59 years 181 days	--	Alive	Mucinous adenocarcinoma	not hispanic or latino	white
TCGA-CD-A486	TCGA-STAD	Stomach	Male	72	9	4	16	11	3	8	16	100	97	2	TCGA	Adenomas and Adenocarcinomas	68 years 42 days	192 days	Dead	Adenocarcinoma, NOS	not hispanic or latino	asian

TCGA-BR-6455	TCGA-STAD	Stomach	Male	74	10	4	16	11	3	8	17	100	97	3	TCGA	Adenomas and Adenocarcinomas	59 years 316 days	1 year 57 days	Dead	Adenocarcinoma, NOS	not hispanic or latino	white
TCGA-BR-8373	TCGA-STAD	Stomach	Female	71	9	4	16	11	3	8	16	96	96	2	TCGA	Adenomas and Adenocarcinomas	65 years 3 days	--	Alive	Adenocarcinoma, intestinal type	not hispanic or latino	white
TCGA-FP-A8CX	TCGA-STAD	Stomach	Male	73	10	4	16	11	3	9	16	96	94	2	TCGA	Cystic, Mucinous and Serous Neoplasms	60 years 85 days	--	Alive	Signet ring cell carcinoma	not hispanic or latino	white

TCGA-BR-4267	TCGA-STAD	Stomach	Male	79	10	4	16	11	6	8	19	94	94	5	TCGA	Adenomas and Adenocarcinomas	51 years 72 days	188 days	Dead	Adenocarcinoma, NOS	not hispanic or latino	white
TCGA-FP-7916	TCGA-STAD	Stomach	Male	71	8	4	16	11	3	8	16	94	93	2	TCGA	Adenomas and Adenocarcinomas	77 years 351 days	1 year 63 days	Dead	Carcinoma, diffuse type	not hispanic or latino	asian
TCGA-BR-A4J8	TCGA-STAD	Stomach	Female	71	8	4	16	11	3	8	16	93	93	2	TCGA	Adenomas and Adenocarcinomas	71 years 116 days	--	Alive	Adenocarcinoma, NOS	not hispanic or latino	white

TCGA-BR-4253	TCGA-STAD	Stomach	Female	79	10	4	16	11	6	8	19	95	93	5	TCGA	Adenomas and Adenocarcinomas	80 years 177 days	124 days	Dead	Adenocarcinoma, NOS	not hispanic or latino	white
TCGA-CG-5717	TCGA-STAD	Stomach	Male	72	10	4	16	9	3	8	17	93	93	3	TCGA	Adenomas and Adenocarcinomas	58 years 274 days	212 days	Dead	Adenocarcinoma, intestinal type	not reported	not reported
TCGA-CG-4474	TCGA-STAD	Stomach	Female	67	9	2	16	11	3	8	17	94	92	3	TCGA	Adenomas and Adenocarcinomas	67 years 215 days	--	Alive	Carcinoma, diffuse type	not reported	not reported

TCGA-BR-A44U	TCGA-STAD	Stomach	Male	74	9	6	16	11	3	8	16	94	92	2	TCGA	Adenomas and Adenocarcinomas	70 years 10 days	1 year 57 days	Dead	Adenocarcinoma, NOS	not hispanic or latino	white
TCGA-B7-A5TN	TCGA-STAD	Stomach	Male	72	10	4	16	11	3	8	15	92	91	1	TCGA	Adenomas and Adenocarcinomas	60 years 47 days	--	Alive	Adenocarcinoma, intestinal type	not hispanic or latino	white
TCGA-D7-A747	TCGA-STAD	Stomach	Male	72	10	4	16	11	3	8	16	92	90	2	TCGA	Adenomas and Adenocarcinomas	57 years 104 days	255 days	Dead	Carcinoma, diffuse type	not hispanic or latino	white

TCGA-VQ-A8P8	TCGA-STAD	Stomach	Female	71	10	4	16	10	3	8	16	91	90	2	TCGA	Adenomas and Adenocarcinomas	72 years 145 days	--	Alive	Adenocarcinoma, intestinal type	not reported	black or african american
TCGA-CD-A4MH	TCGA-STAD	Stomach	Female	71	8	4	16	11	3	8	16	89	89	2	TCGA	Adenomas and Adenocarcinomas	86 years 272 days	--	Alive	Adenocarcinoma, NOS	not hispanic or latino	asian
TCGA-F1-6875	TCGA-STAD	Stomach	Male	74	9	4	16	11	3	8	18	90	89	4	TCGA	Adenomas and Adenocarcinomas	79 years 355 days	6 years 6 days	Dead	Adenocarcinoma, NOS	not hispanic or latino	black or african american

TCGA-KB-A93G	TCGA-STAD	Stomach	Male	69	7	2	16	11	3	9	16	93	89	2	TCGA	Cystic, Mucinous and Serous Neoplasms	68 years 87 days	--	Alive	Signet ring cell carcinoma	not reported	white
TCGA-D7-6820	TCGA-STAD	Stomach	Male	77	12	4	16	11	3	8	18	89	89	4	TCGA	Adenomas and Adenocarcinomas	64 years 110 days	--	Alive	Tubular adenocarcinoma	not hispanic or latino	white
TCGA-CG-5718	TCGA-STAD	Stomach	Female	74	10	4	16	11	3	8	17	88	87	3	TCGA	Adenomas and Adenocarcinomas	78 years 274 days	3 years	Dead	Adenocarcinoma, intestinal type	not reported	not reported

TCGA-BR-8484	TCGA-STAD	Stomach	Male	72	8	4	16	11	3	8	17	88	87	3	TCGA	Adenomas and Adenocarcinomas	61 years 303 days	2 years 36 days	Dead	Adenocarcinoma, NOS	not hispanic or latino	white
TCGA-BR-A4J5	TCGA-STAD	Stomach	Male	78	9	4	18	13	3	8	16	87	86	2	TCGA	Cystic, Mucinous and Serous Neoplasms	56 years 260 days	--	Alive	Mucinous adenocarcinoma	not hispanic or latino	asian
TCGA-CD-5804	TCGA-STAD	Stomach	Male	72	8	4	16	11	3	8	17	86	86	3	TCGA	Adenomas and Adenocarcinomas	90 years	--	Alive	Adenocarcinoma, NOS	not hispanic or latino	asian

TCGA-D7-6524	TCGA-STAD	Stomach	Male	75	10	4	16	11	3	8	18	89	86	4	TCGA	Adenomas and Adenocarcinomas	53 years 174 days	--	Alive	Carcinoma, diffuse type	not hispanic or latino	white
TCGA-D7-8573	TCGA-STAD	Stomach	Male	73	8	4	16	11	3	8	18	87	86	4	TCGA	Adenomas and Adenocarcinomas	57 years 144 days	--	Alive	Tubular adenocarcinoma	not hispanic or latino	white
TCGA-CG-5722	TCGA-STAD	Stomach	Female	86	14	8	16	11	3	8	18	87	85	4	TCGA	Adenomas and Adenocarcinomas	67 years 1 day	--	Alive	Adenocarcinoma, intestinal type	not reported	not reported

TCGA-HU-A4GD	TCGA-STAD	Stomach	Male	69	9	6	16	7	3	8	16	88	85	2	TCGA	Adenomas and Adenocarcinomas	56 years 246 days	--	Alive	Tubular adenocarcinoma	not hispanic or latino	asian
TCGA-HU-A4G6	TCGA-STAD	Stomach	Male	72	10	4	16	11	3	8	16	85	85	2	TCGA	Adenomas and Adenocarcinomas	74 years 180 days	--	Alive	Papillary adenocarcinoma, NOS	not hispanic or latino	asian
TCGA-IN-A7NR	TCGA-STAD	Stomach	Female	72	10	4	16	11	3	8	15	86	84	1	TCGA	Adenomas and Adenocarcinomas	64 years 39 days	--	Alive	Adenocarcinoma, NOS	not hispanic or latino	white

TCGA-D7-A4YX	TCGA-STAD	Stomach	Male	80	11	4	18	13	3	8	16	85	84	2	TCGA	Adenomas and Adenocarcinomas	63 years 239 days	--	Alive	Adenocarcinoma, NOS	not hispanic or latino	white
TCGA-BR-4188	TCGA-STAD	Stomach	Female	70	7	2	16	11	6	8	19	85	84	5	TCGA	Adenomas and Adenocarcinomas	53 years 227 days	226 days	Dead	Adenocarcinoma, NOS	not hispanic or latino	white
TCGA-VQ-AA6B	TCGA-STAD	Stomach	Male	73	10	4	16	11	3	8	16	85	84	2	TCGA	Adenomas and Adenocarcinomas	48 years 320 days	--	Alive	Adenocarcinoma, intestinal type	not reported	white

TCGA-R5-A804	TCGA-STAD	Stomach	Male	60	5	2	16	11	3	8	15	86	84	1	TCGA	Adenomas and Adenocarcinomas	54 years 303 days	140 days	Dead	Carcinoma, diffuse type	not hispanic or latino	black or african american
TCGA-CD-5798	TCGA-STAD	Stomach	Male	74	10	4	16	11	3	8	17	87	84	3	TCGA	Cystic, Mucinous and Serous Neoplasms	82 years 360 days	--	Alive	Mucinous adenocarcinoma	not hispanic or latino	asian
TCGA-BR-6705	TCGA-STAD	Stomach	Female	74	10	4	16	11	3	8	17	85	84	3	TCGA	Adenomas and Adenocarcinomas	68 years 236 days	2 years 49 days	Dead	Adenocarcinoma, NOS	not hispanic or latino	white

TCGA-CG-5720	TCG A- STAD	Stom ach	Male	87	14	8	16	11	3	8	18	83	83	4	TCG A	Aden omas and Aden ocar cino mas	71 years 244 days	30 days	Dead	Aden ocar cino ma, NOS	not repo rted	not repo rted
TCGA-CD-8530	TCG A- STAD	Stom ach	Male	74	10	4	16	11	3	8	17	84	83	3	TCG A	Aden omas and Aden ocar cino mas	52 years	--	Alive	Carci nom a, diffu se type	not hispa nic or latin o	asian
TCGA-BR-8676	TCG A- STAD	Stom ach	Male	74	10	4	16	11	3	8	17	85	83	3	TCG A	Aden omas and Aden ocar cino mas	59 years 70 days	--	Alive	Aden ocar cino ma, intes tinal type	not hispa nic or latin o	whit e

TCGA-RD-A8MW	TCGA-STAD	Stomach	Male	71	8	4	16	11	3	8	16	83	83	2	TCGA	Adenomas and Adenocarcinomas	72 years 292 days	3 years 58 days	Dead	Adenocarcinoma, intestinal type	not hispanic or latino	white
TCGA-CD-A489	TCGA-STAD	Stomach	Male	71	8	4	16	11	3	8	16	84	83	2	TCGA	Adenomas and Adenocarcinomas	58 years 333 days	344 days	Dead	Carcinoma, diffuse type	not hispanic or latino	asian
TCGA-VQ-A8DZ	TCGA-STAD	Stomach	Male	70	8	4	16	11	3	8	16	86	83	2	TCGA	Cystic, Mucinous and Serous Neoplasms	70 years 296 days	1 year 31 days	Dead	Signet ring cell carcinoma	not reported	black or african american

TCGA-CG-4304	TCGA-STAD	Stomach	Male	74	10	4	16	11	3	8	17	84	82	3	TCGA	Adenomas and Adenocarcinomas	84 years 336 days	--	Alive	Adenocarcinoma, NOS	not reported	not reported
TCGA-HF-7136	TCGA-STAD	Stomach	Male	74	10	4	16	11	3	8	17	84	82	3	TCGA	Adenomas and Adenocarcinomas	--	--	Alive	Adenocarcinoma, NOS	not reported	not reported
TCGA-VQ-A94T	TCGA-STAD	Stomach	Male	72	10	4	16	11	3	8	16	81	81	2	TCGA	Adenomas and Adenocarcinomas	73 years 39 days	342 days	Dead	Carcinoma, diffuse type	not reported	not reported

TCGA-D7-6817	TCGA-STAD	Stomach	Male	77	12	4	16	11	3	8	18	83	81	4	TCGA	Adenomas and Adenocarcinomas	63 years 132 days	--	Alive	Tubular adenocarcinoma	not hispanic or latino	white
TCGA-VQ-A8PZ	TCGA-STAD	Stomach	Female	61	6	0	16	11	3	8	16	82	81	2	TCGA	Cystic, Mucinous and Serous Neoplasms	56 years 281 days	--	Alive	Signet ring cell carcinoma	not reported	white
TCGA-VQ-A8PY	TCGA-STAD	Stomach	Female	60	6	0	16	11	3	8	16	81	80	2	TCGA	Adenomas and Adenocarcinomas	47 years 64 days	1 year 71 days	Dead	Tubular adenocarcinoma	not reported	white

TCGA-HF-7133	TCGA-STAD	Stomach	Female	72	8	4	16	11	3	8	17	81	79	3	TCGA	Cystic, Mucinous and Serous Neoplasms	--	--	Alive	Mucinous adenocarcinoma	not reported	not reported
TCGA-VQ-A8DV	TCGA-STAD	Stomach	Male	72	10	4	16	11	3	8	16	78	78	2	TCGA	Adenomas and Adenocarcinomas	48 years 98 days	1 year 38 days	Dead	Adenocarcinoma, intestinal type	not reported	white
TCGA-MX-A663	TCGA-STAD	Stomach	Male	71	8	4	16	11	3	9	16	80	78	2	TCGA	Adenomas and Adenocarcinomas	66 years 315 days	300 days	Dead	Adenocarcinoma, NOS	not hispanic or latino	white

TCGA-CD-8525	TCGA-STAD	Stomach	Female	72	8	4	16	11	3	8	17	79	78	3	TCGA	Cystic, Mucinous and Serous Neoplasms	82 years 54 days	--	Alive	Mucinous adenocarcinoma	not hispanic or latino	asian
TCGA-BR-A4J4	TCGA-STAD	Stomach	Male	80	11	4	18	13	3	8	16	77	77	2	TCGA	Adenomas and Adenocarcinomas	39 years 215 days	--	Alive	Adenocarcinoma, NOS	not hispanic or latino	asian
TCGA-EQ-5647	TCGA-STAD	Stomach	Female	72	10	2	17	14	3	8	18	82	77	4	TCGA	Adenomas and Adenocarcinomas	86 years 20 days	19 days	Dead	Adenocarcinoma, NOS	not hispanic or latino	white

TCGA-IN-7806	TCGA-STAD	Stomach	Male	92	15	8	17	14	3	8	18	76	74	4	TCGA	Adenomas and Adenocarcinomas	50 years 142 days	--	Alive	Carcinoma, diffuse type	not hispanic or latino	white
TCGA-HU-8245	TCGA-STAD	Stomach	Male	69	6	4	17	14	3	8	17	75	74	3	TCGA	Adenomas and Adenocarcinomas	72 years 223 days	--	Alive	Papillary adenocarcinoma, NOS	not hispanic or latino	asian
TCGA-VQ-AA69	TCGA-STAD	Stomach	Male	71	8	4	16	11	3	8	16	74	74	2	TCGA	Adenomas and Adenocarcinomas	35 years 304 days	--	Alive	Adenocarcinoma, NOS	not reported	white

TCGA-R5-A7ZR	TCGA-STAD	Stomach	Female	69	8	4	16	11	3	8	15	74	74	1	TCGA	Adenomas and Adenocarcinomas	70 years 228 days	185 days	Dead	Tubular adenocarcinoma	not hispanic or latino	black or african american
TCGA-BR-8682	TCGA-STAD	Stomach	Male	74	10	4	16	11	3	8	17	74	73	3	TCGA	Cystic, Mucinous and Serous Neoplasms	52 years 172 days	--	Alive	Mucinous adenocarcinoma	not hispanic or latino	asian
TCGA-CG-5734	TCGA-STAD	Stomach	Male	87	14	8	16	11	3	8	18	75	73	4	TCGA	Adenomas and Adenocarcinomas	68 years 242 days	243 days	Dead	Adenocarcinoma, intestinal type	not reported	not reported

TCGA-BR-8677	TCGA-STAD	Stomach	Female	70	8	4	16	9	3	8	17	73	73	3	TCGA	Adenomas and Adenocarcinomas	74 years 195 days	--	Alive	Adenocarcinoma, NOS	not hispanic or latino	white
TCGA-VQ-AA6K	TCGA-STAD	Stomach	Male	73	10	4	16	11	3	8	16	73	73	2	TCGA	Cystic, Mucinous and Serous Neoplasms	59 years 352 days	1 year 13 days	Dead	Signet ring cell carcinoma	not reported	white
TCGA-BR-A4J1	TCGA-STAD	Stomach	Male	78	9	4	18	13	3	8	16	72	72	2	TCGA	Adenomas and Adenocarcinomas	63 years 256 days	22 days	Dead	Adenocarcinoma, NOS	not hispanic or latino	asian

TCGA-BR-A4CR	TCGA-STAD	Stomach	Female	72	9	4	16	11	3	8	16	72	72	2	TCGA	Adenomas and Adenocarcinomas	70 years 102 days	--	Alive	Adenocarcinoma, NOS	not hispanic or latino	white
TCGA-VQ-A94O	TCGA-STAD	Stomach	Male	78	9	4	18	13	3	8	16	72	72	2	TCGA	Adenomas and Adenocarcinomas	74 years 278 days	1 year 275 days	Dead	Adenocarcinoma, intestinal type	not reported	black or african american
TCGA-D7-6518	TCGA-STAD	Stomach	Male	77	12	4	16	11	3	8	18	71	71	4	TCGA	Adenomas and Adenocarcinomas	75 years 201 days	--	Alive	Carcinoma, diffuse type	not hispanic or latino	white

TCGA-D7-A6F2	TCG<br>A-<br>STAD	Stom<br>ach	Male	72	10	4	16	11	3	8	16	71	70	2	TCG<br>A	Aden<br>omas<br>and<br>Aden<br>ocar<br>cino<br>mas	62<br>years<br>156<br>days	--	Alive	Tubu<br>lar<br>aden<br>ocar<br>cino<br>ma	not<br>hispa<br>nic<br>or<br>latin<br>o	whit<br>e
TCGA-BR-8380	TCG<br>A-<br>STAD	Stom<br>ach	Male	72	8	4	16	11	3	8	17	71	70	3	TCG<br>A	Aden<br>omas<br>and<br>Aden<br>ocar<br>cino<br>mas	55<br>years<br>69<br>days	--	Dead	Aden<br>ocar<br>cino<br>ma,<br>NOS	not<br>hispa<br>nic<br>or<br>latin<br>o	whit<br>e
TCGA-BR-6707	TCG<br>A-<br>STAD	Stom<br>ach	Male	76	12	4	16	11	3	8	17	69	69	3	TCG<br>A	Aden<br>omas<br>and<br>Aden<br>ocar<br>cino<br>mas	75<br>years<br>136<br>days	1<br>year<br>240<br>days	Dead	Aden<br>ocar<br>cino<br>ma,<br>NOS	not<br>hispa<br>nic<br>or<br>latin<br>o	whit<br>e

TCGA-RD-A8MV	TCGA-STAD	Stomach	Male	73	10	4	16	11	3	8	16	69	69	2	TCGA	Adenomas and Adenocarcinomas	56 years 317 days	--	Alive	Adenocarcinoma, intestinal type	not hispanic or latino	white
TCGA-KB-A6F5	TCGA-STAD	Stomach	Male	64	7	2	16	11	3	8	16	69	67	2	TCGA	Adenomas and Adenocarcinomas	73 years 218 days	--	Alive	Adenocarcinoma, intestinal type	not reported	not reported
TCGA-BR-7196	TCGA-STAD	Stomach	Male	74	10	4	16	11	3	8	17	67	67	3	TCGA	Adenomas and Adenocarcinomas	64 years 235 days	--	Alive	Adenocarcinoma, NOS	not hispanic or latino	white

TCGA-VQ-A928	TCGA-STAD	Stomach	Male	73	10	4	16	11	3	8	16	66	66	2	TCGA	Adenomas and Adenocarcinomas	49 years 161 days	174 days	Dead	Papillary adenocarcinoma, NOS	not reported	white
TCGA-BR-8296	TCGA-STAD	Stomach	Female	70	8	4	16	9	3	8	17	67	65	3	TCGA	Adenomas and Adenocarcinomas	58 years 343 days	1 year 109 days	Dead	Adenocarcinoma, intestinal type	not hispanic or latino	white
TCGA-D7-6520	TCGA-STAD	Stomach	Male	75	10	4	16	11	3	8	18	65	64	4	TCGA	Adenomas and Adenocarcinomas	53 years 109 days	--	Alive	Tubular adenocarcinoma	not hispanic or latino	white

TCGA-CG-4462	TCGA-STAD	Stomach	Female	73	10	4	16	11	3	8	17	64	64	3	TCGA	Adenomas and Adenocarcinomas	72 years 213 days	--	Dead	Carcinoma, diffuse type	not reported	not reported
TCGA-FP-7998	TCGA-STAD	Stomach	Male	73	9	4	16	11	3	8	17	64	64	3	TCGA	Adenomas and Adenocarcinomas	77 years 178 days	--	Alive	Carcinoma, diffuse type	hispanic or latino	white
TCGA-HU-8610	TCGA-STAD	Stomach	Male	77	9	4	17	14	3	8	17	62	62	3	TCGA	Adenomas and Adenocarcinomas	75 years 201 days	--	Alive	Tubular adenocarcinoma	not hispanic or latino	asian

TCGA-CG-5719	TCGA-STAD	Stomach	Female	74	10	4	16	11	3	8	17	63	62	3	TCGA	Adenomas and Adenocarcinomas	54 years 243 days	--	Alive	Adenocarcinoma, intestinal type	not reported	not reported
TCGA-D7-6521	TCGA-STAD	Stomach	Male	75	10	4	16	11	3	8	18	63	61	4	TCGA	Adenomas and Adenocarcinomas	65 years 35 days	--	Alive	Carcinoma, diffuse type	not hispanic or latino	white
TCGA-BR-8367	TCGA-STAD	Stomach	Male	72	8	4	16	11	3	8	17	63	61	3	TCGA	Adenomas and Adenocarcinomas	55 years 353 days	2 years 71 days	Dead	Adenocarcinoma, NOS	not hispanic or latino	white

TCGA-VQ-A8PD	TCGA-STAD	Stomach	Male	73	10	4	16	11	3	8	16	62	61	2	TCGA	Adenomas and Adenocarcinomas	69 years 42 days	1 year 131 days	Dead	Adenocarcinoma, NOS	not reported	white
TCGA-BR-4255	TCGA-STAD	Stomach	Female	79	10	4	16	11	6	8	19	59	59	5	TCGA	Adenomas and Adenocarcinomas	76 years 134 days	124 days	Dead	Adenocarcinoma, NOS	not hispanic or latino	white
TCGA-BR-6563	TCGA-STAD	Stomach	Male	72	10	4	16	11	3	8	16	59	58	2	TCGA	Adenomas and Adenocarcinomas	60 years 21 days	--	Alive	Adenocarcinoma, NOS	not hispanic or latino	white

TCGA-FP-7735	TCGA-STAD	Stomach	Male	89	15	8	17	13	3	8	17	58	58	3	TCGA	Adenomas and Adenocarcinomas	77 years 108 days	106 days	Dead	Adenocarcinoma, NOS	not hispanic or latino	black or african american
TCGA-D7-8578	TCGA-STAD	Stomach	Male	72	8	4	16	11	3	8	17	57	57	3	TCGA	Adenomas and Adenocarcinomas	72 years 322 days	--	Alive	Adenocarcinoma, NOS	not hispanic or latino	white
TCGA-BR-8295	TCGA-STAD	Stomach	Female	70	8	4	16	9	3	8	17	58	57	3	TCGA	Adenomas and Adenocarcinomas	60 years 341 days	67 days	Dead	Adenocarcinoma, intestinal type	not hispanic or latino	white

TCGA-BR-8486	TCGA-STAD	Stomach	Female	71	9	4	16	11	3	8	15	60	57	1	TCGA	Adenomas and Adenocarcinomas	90 years	--	Alive	Adenocarcinoma, NOS	not hispanic or latino	white
TCGA-R5-A7ZF	TCGA-STAD	Stomach	Female	69	8	4	16	11	3	8	15	57	57	1	TCGA	Adenomas and Adenocarcinomas	65 years 167 days	259 days	Dead	Adenocarcinoma, intestinal type	not hispanic or latino	black or african american
TCGA-CG-5732	TCGA-STAD	Stomach	Male	75	10	4	16	11	3	8	18	57	56	4	TCGA	Adenomas and Adenocarcinomas	66 years 273 days	5 years 274 days	Dead	Adenocarcinoma, intestinal type	not reported	not reported

TCGA-RD-A7BW	TCGA-STAD	Stomach	Female	71	8	4	16	11	3	8	16	58	56	2	TCGA	Adenomas and Adenocarcinomas	68 years 199 days	156 days	Dead	Carcinoma, diffuse type	not hispanic or latino	white
TCGA-CG-4475	TCGA-STAD	Stomach	Male	75	10	4	16	11	3	9	17	56	55	3	TCGA	Adenomas and Adenocarcinomas	76 years 306 days	--	Alive	Adenocarcinoma with mixed subtypes	not reported	not reported
TCGA-HU-A4GY	TCGA-STAD	Stomach	Female	92	14	10	17	14	3	8	17	55	55	3	TCGA	Adenomas and Adenocarcinomas	76 years 354 days	--	Alive	Carcinoma, diffuse type	not hispanic or latino	asian

TCGA-B7-A5TK	TCGA-STAD	Stomach	Male	75	11	6	16	11	3	8	15	55	55	1	TCGA	Adenomas and Adenocarcinomas	51 years 123 days	--	Alive	Adenocarcinoma, intestinal type	not hispanic or latino	white
TCGA-IN-AB1V	TCGA-STAD	Stomach	Male	85	14	8	16	11	3	8	16	57	55	2	TCGA	Adenomas and Adenocarcinomas	63 years 20 days	--	Alive	Adenocarcinoma, NOS	not hispanic or latino	white
TCGA-D7-6519	TCGA-STAD	Stomach	Female	77	12	4	16	11	3	8	18	54	54	4	TCGA	Adenomas and Adenocarcinomas	64 years 37 days	--	Alive	Carcinoma, diffuse type	not hispanic or latino	white

TCGA-VQ-A94P	TCGA-STAD	Stomach	Male	73	10	4	16	11	3	8	16	57	54	2	TCGA	Cystic, Mucinous and Serous Neoplasms	57 years 203 days	81 days	Dead	Signet ring cell carcinoma	not reported	white
TCGA-CD-5802	TCGA-STAD	Stomach	Male	67	9	2	16	11	3	8	17	57	54	3	TCGA	Adenomas and Adenocarcinomas	58 years 147 days	--	Alive	Adenocarcinoma, NOS	not hispanic or latino	asian
TCGA-CG-4301	TCGA-STAD	Stomach	Female	75	11	4	16	11	3	8	17	54	54	3	TCGA	Adenomas and Adenocarcinomas	75 years 123 days	--	Alive	Adenocarcinoma, NOS	not reported	not reported

TCGA-F1-A72C	TCGA-STAD	Stomach	Male	71	8	4	16	11	3	8	16	53	53	2	TCGA	Adenomas and Adenocarcinomas	68 years 267 days	--	Alive	Adenocarcinoma, intestinal type	not hispanic or latino	asian
TCGA-RD-A7BS	TCGA-STAD	Stomach	Male	73	10	4	16	11	3	8	16	52	52	2	TCGA	Adenomas and Adenocarcinomas	46 years 46 days	336 days	Dead	Carcinoma, diffuse type	not hispanic or latino	white
TCGA-BR-4183	TCGA-STAD	Stomach	Female	70	7	2	16	11	6	8	19	52	52	5	TCGA	Adenomas and Adenocarcinomas	55 years 332 days	201 days	Dead	Adenocarcinoma, NOS	not hispanic or latino	white

TCGA-BR-4279	TCGA-STAD	Stomach	Male	79	10	4	16	11	6	8	19	54	52	5	TCGA	Adenomas and Adenocarcinomas	43 years 360 days	291 days	Dead	Adenocarcinoma, NOS	not hispanic or latino	white
TCGA-D7-8575	TCGA-STAD	Stomach	Male	73	8	4	16	11	3	8	18	51	51	4	TCGA	Adenomas and Adenocarcinomas	75 years 319 days	1 year 189 days	Dead	Tubular adenocarcinoma	not hispanic or latino	white
TCGA-VQ-A8PM	TCGA-STAD	Stomach	Male	73	10	4	16	11	3	8	16	53	51	2	TCGA	Cystic, Mucinous and Serous Neoplasms	56 years 211 days	57 days	Dead	Signet ring cell carcinoma	not reported	white

TCGA-HU-A4G2	TCGA-STAD	Stomach	Male	70	8	4	16	11	3	8	16	51	51	2	TCGA	Adenomas and Adenocarcinomas	45 years 355 days	--	Alive	Carcinoma, diffuse type	not hispanic or latino	asian
TCGA-BR-6801	TCGA-STAD	Stomach	Male	76	12	4	16	11	3	8	17	50	50	3	TCGA	Adenomas and Adenocarcinomas	70 years 70 days	--	Alive	Adenocarcinoma, NOS	not hispanic or latino	white
TCGA-CD-8526	TCGA-STAD	Stomach	Female	74	10	4	16	11	3	8	17	50	50	3	TCGA	Adenomas and Adenocarcinomas	73 years 177 days	--	Alive	Adenocarcinoma, intestinal type	not hispanic or latino	asian

TCGA-ZA-A8F6	TCGA-STAD	Stomach	Male	73	10	4	16	11	3	8	16	48	48	2	TCGA	Adenomas and Adenocarcinomas	71 years 190 days	--	Alive	Adenocarcinoma, intestinal type	not hispanic or latino	white
TCGA-VQ-A91Y	TCGA-STAD	Stomach	Male	73	10	4	16	11	3	8	16	48	48	2	TCGA	Adenomas and Adenocarcinomas	67 years 303 days	296 days	Dead	Tubular adenocarcinoma	not reported	white
TCGA-VQ-AA6F	TCGA-STAD	Stomach	Male	73	10	4	16	11	3	8	16	49	48	2	TCGA	Adenomas and Adenocarcinomas	57 years 351 days	--	Alive	Adenocarcinoma, NOS	not reported	white

TCGA-VQ-A8PS	TCGA-STAD	Stomach	Male	59	4	0	16	11	3	8	16	49	48	2	TCGA	Adenomas and Adenocarcinomas	76 years 276 days	1 year 41 days	Dead	Adenocarcinoma, intestinal type	not reported	white
TCGA-BR-A453	TCGA-STAD	Stomach	Male	69	6	4	16	11	3	8	16	47	47	2	TCGA	Cystic, Mucinous and Serous Neoplasms	51 years 361 days	185 days	Dead	Signet ring cell carcinoma	not hispanic or latino	asian
TCGA-BR-6456	TCGA-STAD	Stomach	Female	82	13	4	17	14	3	8	18	47	47	4	TCGA	Adenomas and Adenocarcinomas	74 years 260 days	1 year 161 days	Dead	Adenocarcinoma, NOS	not hispanic or latino	white

TCGA-CG-4455	TCGA-STAD	Stomach	Male	65	7	2	16	11	3	8	17	47	46	3	TCGA	Cystic, Mucinous and Serous Neoplasms	72 years 123 days	--	Alive	Mucinous adenocarcinoma	not reported	not reported
TCGA-VQ-A91W	TCGA-STAD	Stomach	Male	72	9	4	16	11	3	8	16	45	44	2	TCGA	Adenomas and Adenocarcinomas	30 years 6 days	--	Alive	Adenocarcinoma, intestinal type	not reported	white
TCGA-BR-8058	TCGA-STAD	Stomach	Female	70	8	4	16	11	3	8	15	44	44	1	TCGA	Adenomas and Adenocarcinomas	53 years 300 days	--	Alive	Adenocarcinoma, NOS	not hispanic or latino	white

TCGA-CD-8532	TCGA-STAD	Stomach	Male	72	8	4	16	11	3	8	17	44	44	3	TCGA	Adenomas and Adenocarcinomas	52 years 291 days	354 days	Dead	Adenocarcinoma, NOS	not hispanic or latino	asian
TCGA-BR-7957	TCGA-STAD	Stomach	Female	78	9	4	17	14	3	8	18	45	44	4	TCGA	Adenomas and Adenocarcinomas	50 years 255 days	276 days	Dead	Adenocarcinoma, intestinal type	not hispanic or latino	white
TCGA-BR-8384	TCGA-STAD	Stomach	Male	71	8	4	16	11	3	8	16	45	43	2	TCGA	Adenomas and Adenocarcinomas	69 years 79 days	--	Alive	Adenocarcinoma, NOS	not hispanic or latino	white

TCGA-BR-7722	TCGA-STAD	Stomach	Male	73	10	4	16	11	3	8	16	44	43	2	TCGA	Adenomas and Adenocarcinomas	62 years 56 days	1 year 101 days	Dead	Adenocarcinoma, NOS	not hispanic or latino	white
TCGA-VQ-A927	TCGA-STAD	Stomach	Male	71	10	4	16	10	3	8	16	43	43	2	TCGA	Adenomas and Adenocarcinomas	81 years 91 days	200 days	Dead	Carcinoma, diffuse type	not reported	white
TCGA-BR-A4J7	TCGA-STAD	Stomach	Male	78	9	4	18	13	3	8	16	42	42	2	TCGA	Cystic, Mucinous and Serous Neoplasms	49 years 35 days	--	Alive	Mucinous adenocarcinoma	not hispanic or latino	asian

TCGA-BR-A4J2	TCGA-STAD	Stomach	Male	78	9	4	18	13	3	8	16	39	39	2	TCGA	Adenomas and Adenocarcinomas	70 years 324 days	--	Alive	Carcinoma, diffuse type	not hispanic or latino	asian
TCGA-D7-A748	TCGA-STAD	Stomach	Female	73	10	4	16	11	3	8	16	39	39	2	TCGA	Adenomas and Adenocarcinomas	41 years 215 days	132 days	Dead	Carcinoma, diffuse type	not hispanic or latino	white
TCGA-BR-A4J6	TCGA-STAD	Stomach	Female	78	9	4	18	13	3	8	16	36	36	2	TCGA	Adenomas and Adenocarcinomas	69 years 77 days	--	Alive	Adenocarcinoma, NOS	not hispanic or latino	white

TCGA-BR-6709	TCGA-STAD	Stomach	Female	80	11	4	17	14	3	8	18	38	36	4	TCGA	Adenomas and Adenocarcinomas	57 years 116 days	1 year 5 days	Dead	Adenocarcinoma, NOS	not hispanic or latino	white
TCGA-CD-5803	TCGA-STAD	Stomach	Female	74	10	4	16	11	3	8	17	34	34	3	TCGA	Adenomas and Adenocarcinomas	78 years 119 days	341 days	Dead	Adenocarcinoma, NOS	not hispanic or latino	asian
TCGA-MX-A5UG	TCGA-STAD	Stomach	Male	73	10	4	16	11	3	8	16	34	34	2	TCGA	Adenomas and Adenocarcinomas	78 years 225 days	113 days	Dead	Carcinoma, diffuse type	not hispanic or latino	asian

TCGA-CD-5799	TCGA-STAD	Stomach	Male	74	12	4	16	9	3	8	17	35	34	3	TCGA	Adenomas and Adenocarcinomas	45 years 97 days	--	Alive	Adenocarcinoma, intestinal type	not hispanic or latino	asian
TCGA-BR-4294	TCGA-STAD	Stomach	Male	79	10	4	16	11	6	8	19	33	33	5	TCGA	Adenomas and Adenocarcinomas	65 years 17 days	--	Alive	Tubular adenocarcinoma	not hispanic or latino	white
TCGA-D7-8574	TCGA-STAD	Stomach	Male	72	8	4	16	11	3	8	17	32	32	3	TCGA	Adenomas and Adenocarcinomas	72 years 288 days	--	Alive	Adenocarcinoma, NOS	not hispanic or latino	white

TCGA-BR-8592	TCGA-STAD	Stomach	Female	72	8	4	16	11	3	8	17	30	30	3	TCGA	Adenomas and Adenocarcinomas	63 years 123 days	191 days	Dead	Carcinoma, diffuse type	not hispanic or latino	white
TCGA-BR-8291	TCGA-STAD	Stomach	Male	72	8	4	16	11	3	8	17	30	30	3	TCGA	Adenomas and Adenocarcinomas	61 years 272 days	1 year 242 days	Dead	Carcinoma, diffuse type	not hispanic or latino	white
TCGA-RD-A8N5	TCGA-STAD	Stomach	Male	68	10	4	16	7	3	8	16	29	29	2	TCGA	Cystic, Mucinous and Serous Neoplasms	78 years 100 days	4 years 286 days	Dead	Mucinous adenocarcinoma	not hispanic or latino	white

TCGA-D7-6522	TCGA-STAD	Stomach	Male	75	10	4	16	11	3	8	18	28	28	4	TCGA	Adenomas and Adenocarcinomas	58 years 281 days	--	Alive	Carcinoma, diffuse type	not hispanic or latino	white
TCGA-BR-6564	TCGA-STAD	Stomach	Female	94	17	8	17	14	3	8	18	28	28	4	TCGA	Adenomas and Adenocarcinomas	46 years 328 days	2 years 64 days	Dead	Adenocarcinoma, NOS	not hispanic or latino	white
TCGA-BR-8365	TCGA-STAD	Stomach	Female	71	8	4	16	11	3	8	16	25	25	2	TCGA	Adenomas and Adenocarcinomas	70 years 158 days	1 year 168 days	Dead	Adenocarcinoma, NOS	not hispanic or latino	white

TCGA-D7-A74B	TCGA-STAD	Stomach	Female	62	7	2	16	10	3	8	16	25	24	2	TCGA	Adenomas and Adenocarcinomas	52 years 73 days	217 days	Dead	Carcinoma, diffuse type	not hispanic or latino	white
TCGA-BR-4187	TCGA-STAD	Stomach	Male	79	10	4	16	11	6	8	19	21	21	5	TCGA	Adenomas and Adenocarcinomas	56 years 181 days	141 days	Dead	Adenocarcinoma, NOS	not hispanic or latino	white
TCGA-BR-A4IU	TCGA-STAD	Stomach	Female	71	8	4	16	11	3	8	16	21	21	2	TCGA	Adenomas and Adenocarcinomas	34 years 183 days	--	Alive	Carcinoma, diffuse type	not hispanic or latino	asian

TCGA-D7-A6ET	TCGA-STAD	Stomach	Male	63	7	2	16	11	3	8	16	21	21	2	TCGA	Adenomas and Adenocarcinomas	76 years 169 days	--	Alive	Tubular adenocarcinoma	not hispanic or latino	white
TCGA-BR-8371	TCGA-STAD	Stomach	Male	72	8	4	16	11	3	8	17	19	19	3	TCGA	Adenomas and Adenocarcinomas	62 years 238 days	359 days	Dead	Adenocarcinoma, NOS	not hispanic or latino	white
TCGA-BR-8364	TCGA-STAD	Stomach	Female	72	8	4	16	11	3	8	17	16	16	3	TCGA	Adenomas and Adenocarcinomas	42 years 49 days	--	Alive	Carcinoma, diffuse type	not hispanic or latino	white

TCGA-BR-A4IV	TCGA-STAD	Stomach	Male	71	8	4	16	11	3	8	16	16	16	2	TCGA	Adenomas and Adenocarcinomas	47 years 349 days	2 years 139 days	Dead	Carcinoma, diffuse type	not hispanic or latino	asian
TCGA-HU-A4HB	TCGA-STAD	Stomach	Male	90	15	8	17	14	3	8	17	14	14	3	TCGA	Adenomas and Adenocarcinomas	68 years 294 days	1 year 112 days	Dead	Carcinoma, diffuse type	not hispanic or latino	asian
TCGA-BR-A44T	TCGA-STAD	Stomach	Female	63	8	4	15	4	3	8	16	14	14	2	TCGA	Adenomas and Adenocarcinomas	53 years 49 days	--	Alive	Carcinoma, diffuse type	not hispanic or latino	white

TCGA-BR-6803	TCGA-STAD	Stomach	Female	82	13	4	17	14	3	8	18	13	13	4	TCGA	Adenomas and Adenocarcinomas	54 years 28 days	--	Alive	Carcinoma, diffuse type	not hispanic or latino	white
TCGA-CG-5716	TCGA-STAD	Stomach	Male	71	8	4	16	10	3	9	16	9	9	2	TCGA	Adenomas and Adenocarcinomas	86 years	--	Alive	Adenocarcinoma, NOS	not reported	not reported
TCGA-VQ-A8PQ	TCGA-STAD	Stomach	Female	73	10	4	16	11	3	8	16	8	8	2	TCGA	Adenomas and Adenocarcinomas	50 years 56 days	1 year 111 days	Dead	Tubular adenocarcinoma	not reported	white

TCGA-BR-A4IZ	TCGA-STAD	Stomach	Female	71	8	4	16	11	3	8	16	8	8	2	TCGA	Adenomas and Adenocarcinomas	45 years 259 days	273 days	Dead	Carcinoma, diffuse type	not hispanic or latino	asian
TCGA-HU-A4GJ	TCGA-STAD	Stomach	Female	71	10	4	16	10	3	8	16	4	4	2	TCGA	Adenomas and Adenocarcinomas	60 years 164 days	--	Alive	Carcinoma, diffuse type	not hispanic or latino	asian
TCGA-FP-8210	TCGA-STAD	Stomach	Male	69	8	4	16	11	3	8	15	3	3	1	TCGA	Adenomas and Adenocarcinomas	48 years 30 days	153 days	Dead	Carcinoma, diffuse type	not hispanic or latino	asian

TCGA-RD-A8N0	TCGA-STAD	Stomach	Female	72	10	4	16	10	3	8	16	2	2	2	TCGA	Adenomas and Adenocarcinomas	53 years 133 days	--	Alive	Carcinoma, diffuse type	not hispanic or latino	white
TCGA-RD-A8N4	TCGA-STAD	Stomach	Female	70	8	4	16	11	3	8	16	1	1	2	TCGA	Adenomas and Adenocarcinomas	58 years 140 days	--	Alive	Carcinoma, diffuse type	not hispanic or latino	white
TCGA-D7-8576	TCGA-STAD	Stomach	Female	67	8	4	14	9	3	8	17	0	0	3	TCGA	Adenomas and Adenocarcinomas	54 years 291 days	1 year 81 days	Dead	Tubular adenocarcinoma	not hispanic or latino	white

TCGA-CG-4472	TCGA-STAD	Stomach	Male	50	6	2	2	11	3	8	17	0	0	3	TCGA	Adenomas and Adenocarcinomas	49 years 213 days	--	Alive	Adenocarcinoma with mixed subtypes	not reported	not reported
TCGA-BR-8362	TCGA-STAD	Stomach	Male	72	10	4	14	11	3	8	17	0	0	3	TCGA	Adenomas and Adenocarcinomas	65 years 56 days	1 year 33 days	Dead	Adenocarcinoma, NOS	not hispanic or latino	white
TCGA-CD-8535	TCGA-STAD	Stomach	Male	70	8	4	14	11	3	8	17	0	0	3	TCGA	Adenomas and Adenocarcinomas	59 years 10 days	--	Alive	Adenocarcinoma, NOS	not hispanic or latino	asian

TCGA-BR-4367	TCGA-STAD	Stomach	Male	64	9	4	2	11	6	8	19	0	0	5	TCGA	Adenomas and Adenocarcinomas	78 years 328 days	--	Alive	Adenocarcinoma, NOS	not hispanic or latino	white
TCGA-BR-6710	TCGA-STAD	Stomach	Male	89	13	4	19	16	3	8	19	0	0	5	TCGA	Adenomas and Adenocarcinomas	41 years 341 days	--	Alive	Adenocarcinoma, NOS	not hispanic or latino	white
TCGA-RD-A8N2	TCGA-STAD	Stomach	Female	71	10	4	16	10	3	8	16	0	0	2	TCGA	Adenomas and Adenocarcinomas	59 years 200 days	--	Alive	Carcinoma, diffuse type	not hispanic or latino	white

TCGA-BR-8679	TCGA-STAD	Stomach	Female	68	8	4	14	9	3	8	17	0	0	3	TCGA	Adenomas and Adenocarcinomas	63 years 74 days	--	Alive	Adenocarcinoma, NOS	not hispanic or latino	white
TCGA-FP-8209	TCGA-STAD	Stomach	Male	69	8	4	16	10	3	8	16	0	0	2	TCGA	Adenomas and Adenocarcinomas	49 years 274 days	4 years 350 days	Dead	Carcinoma, diffuse type	not hispanic or latino	white
TCGA-BR-A4J9	TCGA-STAD	Stomach	Male	70	9	4	14	11	3	8	16	0	0	2	TCGA	Adenomas and Adenocarcinomas	55 years 5 days	--	Alive	Adenocarcinoma, NOS	not hispanic or latino	white

TCGA-CD-8534	TCGA-STAD	Stomach	Male	68	8	4	14	9	3	8	17	0	0	3	TCGA	Adenomas and Adenocarcinomas	41 years 252 days	--	Alive	Carcinoma, diffuse type	not hispanic or latino	asian
TCGA-BR-8289	TCGA-STAD	Stomach	Male	68	8	4	14	9	3	8	17	0	0	3	TCGA	Adenomas and Adenocarcinomas	57 years 30 days	81 days	Dead	Adenocarcinoma, intestinal type	not hispanic or latino	white

Table S4 Different macrophage subtype markers.

Phenotype	Stimuli	Cell expression markers	Cytokines,chemokines,and other secreted mediators
M1	IFNγ,TNFα,LPS	CD80,CD86,CD68,IL1R1,TLR2,TLR4,NOS2,IL10↑	TNF,IL1B,IL6,IL27,CXCL9,CXCL10,CXCL11,CXCL16,CXCL5
M2a	IL4,IL13	MRC1,IL1RN,IL1R2	IL10,CCL17,CCL18,CCL22,CCL24
M2b	IL1β,TLR	IL10↑,CD86	TNF,IL1B,IL6,IL10,CCL1
M2c	IL10,TGFβ	MRC1,TLR1,TLR8	IL10,CCL16,CCL18,CXCL13
M2d	TLR	VEGFA,TNF↓,IL10↑	IL10,VEGFA
